# Review on Cathode‐Electrolyte Interphase for Stabilizing Interfaces in Solid‐State Lithium Batteries

**DOI:** 10.1002/advs.202517032

**Published:** 2025-11-12

**Authors:** Xinchao Hu, Hongfei Zheng, Chengkun Zhang, Shiyu Zhang, Yuming Jin, Dong‐Liang Peng, Qingshui Xie

**Affiliations:** ^1^ State Key Laboratory of Physical Chemistry of Solid Surface Fujian Key Laboratory of Surface and Interface Engineering for High Performance Materials College of Materials Xiamen University Xiamen Fujian 361005 China; ^2^ Fujian Provincial Key Laboratory of Functional Materials and Applications Institute of Advanced Energy Materials School of Materials Science and Engineering Xiamen University of Technology Xiamen 361024 China

**Keywords:** cathode‐electrolyte interphase, CEI regulation, lithium batteries, solid‐state electrolyte

## Abstract

Solid‐state lithium batteries (SSLBs) hold great promise for improving battery safety and achieving higher energy density. Nevertheless, the matching diversity between solid‐state electrolytes (SEs) and the paired high‐voltage cathodes complicate to understand the formation and failure mechanisms of cathode‐electrolyte interphase (CEI). Furthermore, the structural degradation of cathodes and the electrochemical decomposition of SEs during cycling continuously reconfigure the CEI and alter the CEI composition and structure, adding further complexity to its analysis. The limited understanding of these processes hinders the development of effective strategies to address persistent cathode‐electrolyte interfacial issues (e.g., high interfacial impedance and contact loss) that shorten SSLB lifetime. A prerequisite for overcoming these challenges is to establish a comprehensive knowledge of CEI properties, which remains elusive and is often underestimated. Therefore, this review provides a comprehensive overview of the mechanisms governing CEI formation and failure, elucidating the complex nature of the interphase. Key challenges related to interfacial stability are discussed in detail, along with corresponding strategies for CEI regulation. Innovatively, an expanded conceptual CEI framework is proposed based on recent advances, facilitating a deeper understanding of CEI properties. Finally, forward‐looking perspectives are provided for designing stable CEIs and further advancing SSLB technology.

## Graphical abstract

The cathode‐electrolyte interphase in solid‐state batteries faces new issues due to the solid nature of the electrolyte, including poor self‐repair of CEI, insufficient interface passivation, physical contact loss, and inferior lithium ion transport kinetics. These issues is examined from a broader perspective, promoting the proposal of a generalized CEI to solid‐state batteries.

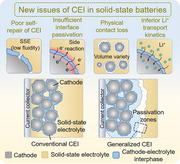

## 1 Introduction

Rechargeable lithium‐ion batteries (LIBs) with high energy density and durable cyclability have shaped our everyday life and promoted the rapid development of consumer electronics and electric vehicles.^[^ ^1^, ^2^, ^3^, ^4^, ^5^, ^6^ ^]^ Some major roadmaps, including China Battery Roadmap and US Battery500,^[^ ^7^ ^]^ have set specified targets for next‐generation LIB technologies, aiming for higher energy density (e.g., 500 Wh kg^−1^) and enhanced safety.^[^ ^8^, ^9^ ^]^ In this regard, solid‐state lithium batteries (SSLBs) emerge as promising candidates that can break through the safety and energy‐density bottlenecks in conventional liquid LIBs. As the core component of SSLBs, the solid‐state electrolytes (SEs) functionally replace the separator and liquid electrolyte (LE), and their characteristics of low flammability,^[^ ^10^, ^11^ ^]^ no leakage risks, and higher thermal stability contribute to reducing the risks of fire and explosion, attracting wide research attention.^[^ ^12^, ^13^, ^14^, ^15^, ^16^ ^]^ Currently, various advanced SEs, primarily divided into two categories, i,e., polymer electrolytes (e.g., solid polymer electrolytes and gel polymer electrolytes) and inorganic solid electrolytes (e.g., oxides, sulfides, and halides), have been designed with the expectation of achieving high ionic conductivities and high safety.

Apart from the development of high‐performance SEs, pursuing higher energy density in SSLBs system also requires the adoption of high‐capacity cathode and anode materials, such as high‐voltage lithium cobalt oxide, Ni‐rich layered oxides, Li‐rich layered oxides, and lithium metal anode.^[^ ^17^, ^18^ ^]^ Yet these high‐voltage and high‐capacity SSLBs systems trigger exceptionally severe and complex interfacial side reactions between SEs and electrode materials,^[^ ^19^ ^]^ which can be broadly classified into two categories: i) the electrochemical decomposition of SEs in contact with conductive components if exceeding their stable voltage limit and ii) spontaneous chemical reactions driven by mismatched chemical potentials between SEs and active materials.^[^ ^20^, ^21^ ^]^ The resulting interfacial issues, such as high interfacial resistance and inhomogeneous ion transport,^[^ ^22^, ^23^ ^]^ greatly hinders the interfacial kinetics of lithium ions and undermines the electrochemical performance, remaining a primary roadblock to practical SSLB deployment. Resolving these challenges requires, first and foremost, a fundamental understanding of how electrode‐electrolyte interphases (EEI) form, evolve, and ultimately fail upon cycling, followed by rationally designed strategies to construct robust and uniform EEI. However, this task is complicated by the diversity of SE species (i.e., oxides, sulfides, halides, and polymers) and their diverse crystal structures and chemical/electrochemical properties (e.g., different electrochemical stability windows and distinct reactive routes), obscuring universal design rules for stable EEI. Particularly, under such high‐voltage circumstances, the cathode materials inevitably release lattice oxygen,^[^ ^24^ ^]^ undergo phase transitions,^[^ ^25^ ^]^ and present highly reactive surfaces,^[^ ^26^ ^]^ which degrade the cathode‐electrolyte interphase (CEI) or attack adjacent SEs. These processes continuously reconfigure the CEI and alert its composition and structure, which adds further complexity to CEI analysis and its structure, as well as raising the susceptibility of CEI breakdown and the electrochemical decay of SSLBs. Therefore, a comprehensive knowledge of the CEI structure and property is indispensable for fundamentally enhancing the performance of SSLBs, while it remains obscure and even often being underestimated.

**Figure 1:**
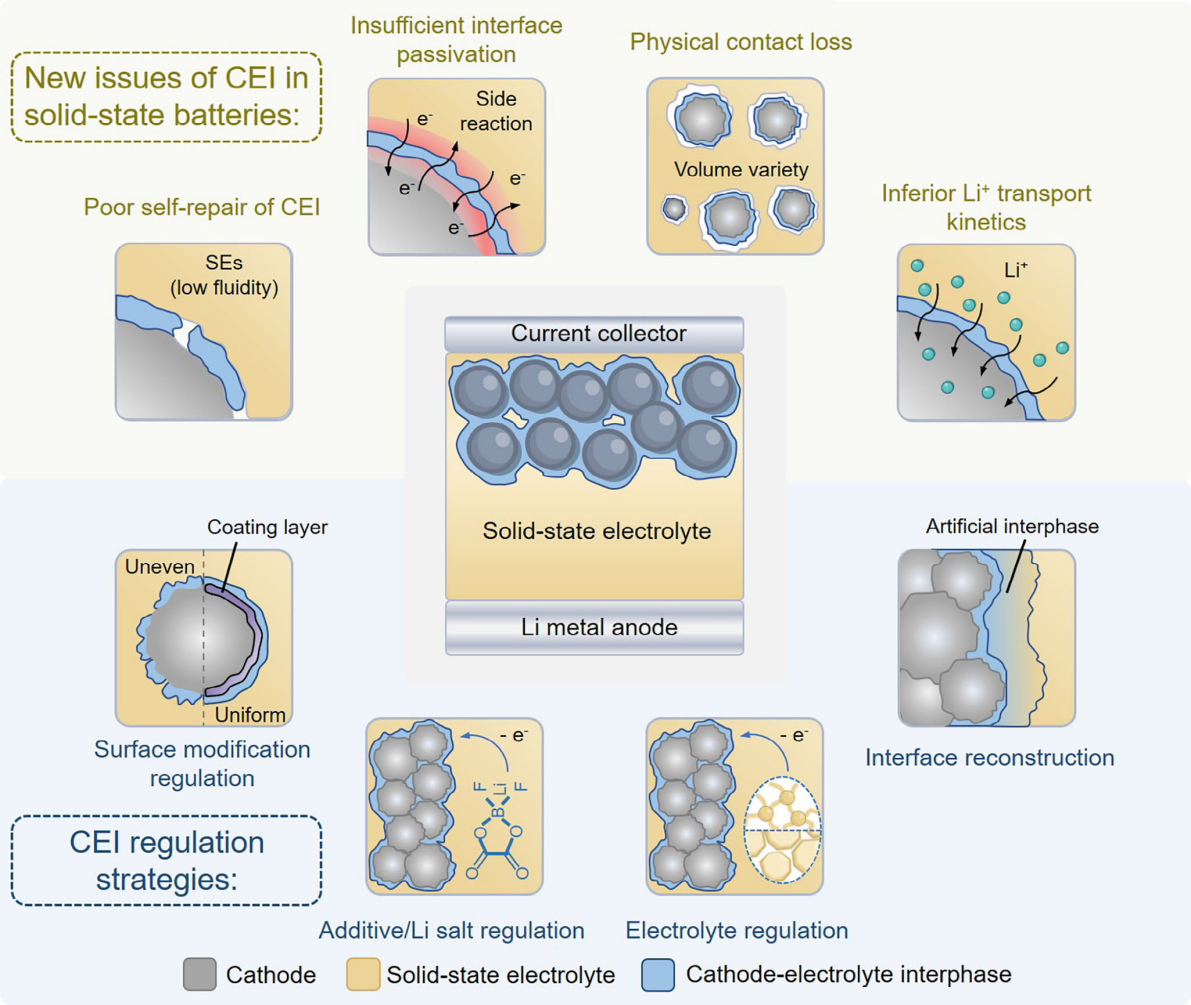
Schematic illustration of new issues of cathode‐electrolyte interphase and regulation strategies in solid‐state batteries.

This review provides a comprehensive overview of the mechanisms governing CEI formation and failure, elucidating the complex nature of the interphase that is rooted in the varied characteristics of SEs and their dynamic interactions with different cathodes. Furthermore, key challenges related to the cathode‐electrolyte interfacial stability are discussed in detail, along with corresponding regulation strategies for CEI modulation (**Figure** **1**). We also thoroughly summarize the essential features and emerging requirements for CEI in future SSLBs. Innovatively, an expanded conceptual CEI framework is proposed based on recent advances, facilitating a deeper understanding of CEI properties and functions. Subsequently, following a throughout understanding of CEI‐related mechanisms and advances, forward‐looking perspectives are provided for designing stable CEIs and further advancing SSLB technology.

## 2 Mini Overview: Fundamentals of CEI from Liquid LIBs to SSLBs

The underlying mechanisms and properties of CEI have been studied for many years and its importance and functions have been highly recognized. The first report on the CEI appeared in 1985, identifying a polymer interphase on the surface of LiCoO_2_ (LCO) cathodes that closely resembles the solid electrolyte interphase (SEI).^[^ ^27^, ^28^ ^]^ Subsequent studies have demonstrated CEI formation on various cathodes like transition metal oxides. The thickness of the formed CEI layer ranges from several to tens of nanometers,^[^ ^29^, ^30^, ^31^ ^]^ which (or whose composition and thickness) is strongly influenced by types of cathode materials, electrolyte solvent, lithium salt, and additives.^[^ ^32^, ^33^ ^]^

### 2.1 Formation Mechanisms and Composition of CEI in Liquid LIBs

Similar to the SEI at the anode side, CEI plays a crucial role in ensuring the electrochemical stability and reversibility in conventional liquid LIBs. CEI can stabilize the cathode‐electrolyte interface and maintain the structural and mechanical integrity of cathode particles by suppressing side reactions, mitigating acid‐induced erosion, and suppressing the dissolution of transition metals (TMs), which enhances battery cyclability.^[^ ^5^, ^34^, ^35^ ^]^ Various advanced characterization techniques, including X‐ray absorption spectroscopy (XAS),^[^ ^36^ ^]^ Fourier transform infrared spectroscopy (FTIR),^[^ ^37^ ^]^ time‐of‐flight secondary ion mass spectroscopy (TOF‐SIMS),^[^ ^38^ ^]^ X‐ray photoelectron spectroscopy (XPS),^[^ ^39^ ^]^ and Cryogenic transmission electron microscopy (Cryo‐TEM),^[^ ^40^ ^]^ have been widely employed to reveal CEI formation in liquid LIBs, where two kinds of mechanisms have emerged. One proposes the in situ formation of CEI through direct electrochemical reactions between the cathode and the liquid electrolyte.^[^ ^41^ ^]^ Alternatively, another theory suggests that CEI formation is influenced by chemical crosstalk of SEI at the anode side. The SEI decomposition fragments migrate through the electrolyte and subsequently deposit on the cathode surface, thus affecting the composition and properties of CEI.^[^ ^42^, ^43^, ^44^ ^]^ Generally, in conventional carbonate‐based electrolytes, the CEI is widely described as a bilayer structure, consisting of an inner layer dominated by inorganic compounds such as LiF and an outer layer of organic species like RCO_x_Li.^[^ ^5^, ^45^, ^46^, ^47^, ^48^ ^]^ Furthermore, the CEI composition is complex, featuring other components such as Li_2_CO_3_, Li_2_O, Li_x_PO_y_F_z_, and TMF_n_ without an established pattern throughout the CEI.^[^ ^49^, ^50^, ^51^, ^52^, ^53^ ^]^ It varies depending on the actual electrolyte formulation, electrode properties, battery operating environment, and testing protocols.^[^ ^18^ ^]^

### 2.2 Characteristics of CEI Required for SSLBs

Although many relevant research have been reported for interface stability in SSLBs, the significance of CEI is still underestimated. The interfacial challenges at the cathode side in SSLBs are governed by two distinct characteristics. First, unlike LEs, SEs possess virtually no fluidity, thus they cannot flow to re‐establish intimate cathode‐electrolyte contact once the microscopic voids generate during cycling due to the repeated volumetric expansion and contraction of the composite cathode.^[^ ^54^ ^]^ This drives a rapid and continuous rise in interfacial resistance, which ultimately degrades the electrochemical performance of SSLBs.^[^ ^55^ ^]^ Second, SEs often exhibit electrochemical and chemical instability when in contact with the cathode, which is not only accelerated by the electrocatalytic effect of the transition metal from the cathode,^[^ ^56^ ^]^ but also triggered spontaneously upon contact with the cathode materials.^[^ ^57^ ^]^ To address the aforementioned issue, the ideal CEI must passivate the interface to prevent further SE degradation, along with its formation of a thin, uniform, intact, and lithium ion‐conductive sheath that enables rapid lithium ion transfer between the electrolyte and the active particles and continuous ionic pathways across the limited solid–solid contact area.^[^ ^22^, ^58^ ^]^ Moreover, the CEI must be electrically insulating to guarantee electrochemical stability while exhibiting sufficient toughness and strength to buffer repeated cathode volume changes, thereby lowering the stack pressure required for SSLBs. These requirements diverge sharply from those of conventional liquid‐based battery systems.

Additionally, the numerous types of electrolytes, such as gel polymer electrolytes (GPEs), solid polymer electrolytes (SPEs), sulfide/halide electrolytes (S/HEs), and oxide electrolytes (OEs), lead to a great difference in CEI constituents. This is particularly evident in multi‐elements electrolytes, whose constituent elements are inevitably incorporated into chemical compositions of the CEI, thereby exacerbating the unpredictability of its properties and function. Conversely, in SSLBs, the low TM solubility of SEs significantly suppresses TM dissolution from the cathode,^[^ ^59^, ^60^, ^61^ ^]^ thus reducing both TM contamination of the CEI and the catalytic decomposition of SE by TM.

### 2.3 Generalized CEI

**Figure 2:**
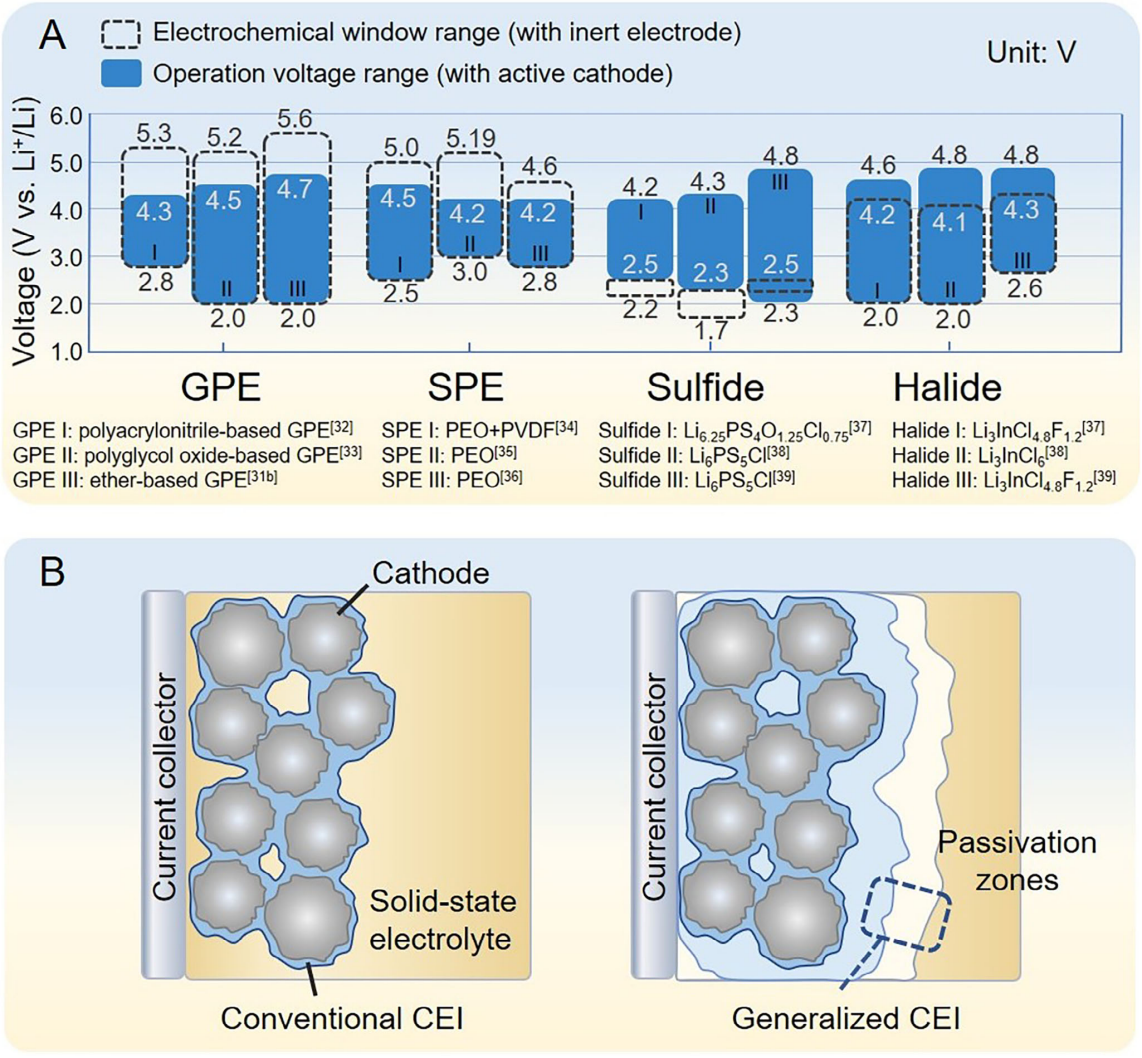
A) The electrochemical window of various SEs measured with an inert electrode and the practical operation voltage range for various SSLBs. The representative electrolyte compositions: GPE (polyacrylonitrile‐based GPE,^[^ ^62^ ^]^ polyglycol oxide‐based GPE,^[^ ^63^ ^]^ ether‐based GPE^[^ ^60^ ^]^), SPE (PEO+PVDF,^[^ ^64^ ^]^ PEO,^[^ ^65^ ^]^ PEO^[^ ^66^ ^]^), sulfide (Li_6.25_PS_4_O_1.25_Cl_0.75_,^[^ ^67^ ^]^ Li_6_PS_5_Cl,^[^ ^68^ ^]^ Li_6_PS_5_Cl^[^ ^69^ ^]^), halide (Li_3_InCl_4.8_F_1.2_,^[^ ^12^ ^]^ Li_3_InCl_6_,^[^ ^70^ ^]^ Li_3_InCl_4.8_F_1.2_^[^ ^71^ ^]^). B) Schematic diagram of conventional and generalized CEI.

It is a common phenomenon that an obvious discrepancy exists between the electrochemical windows of SEs measured on inert electrodes (e.g., stainless steel) and the practical voltage range in SSLBs (**Figure** **2**A). For example, the SPEs (e.g., typical polyethylene oxide, PEO) exhibit a lower oxidative stability voltage than their intrinsic value due to the catalytic effect of the active cathode and conductive additives, which promote side reactions so that the resulting CEI fails to passivate.^[^ ^72^, ^73^ ^]^ Conversely, another type of such discrepancy is that SEs with a narrow electrochemical window (e.g., sulfides) unexpectedly demonstrate stable electrochemical performance with high‐voltage cathodes (>4.5 V).^[^ ^69^, ^74^ ^]^ To rationalize this anomalous voltage tolerance and other CEI‐related phenomena discussed below, we propose a broader interpretation of the CEI in SSLBs. In liquid LIBs, CEI is generally defined as the layer of electrolyte‐decomposition products that adsorbs onto the cathode surface. Since the liquid electrolyte lacks anchoring sites, no analogous interphase forms on its side. However, due to the physical properties of SEs, CEI or CEI‐analog layers can form not only on the electrode but also on the electrolyte surfaces in SSLBs. Furthermore, the crosstalk effect of SEI on CEI is also largely eliminated due to the isolation of SEs.^[^ ^75^ ^]^ Hence, the different CEI formation mechanism results in entirely distinct scopes of CEI definition.^[^ ^40^ ^]^ When the highly reactive sulfides are employed, such CEI generates at the cathode and electrolyte interface. Meanwhile, the decomposition of bulk electrolyte continues to occur during the initial few cycles, forming degraded SE passivation zones (consisting of polysulfide, phosphate, sulfates, and sulfites) whose functions resemble those of CEI at the localized interface (Figure 2B).^[^ ^13^ ^]^ Here, we term the interfacial CEI and these degraded SE zones as the generalized CEI whose formation mechanisms and regulation strategies are fundamentally different conventional CEI. This generalized CEI constitutes a large‐scale chemical transition layer, whose composition evolves from chemically stable decomposition products at the cathode surface to species approaching the pristine electrolyte deeper within the SE, thereby enabling compatibility between high‐voltage cathodes and highly reactive solid electrolytes. More broadly, the generalized CEI should include all interphases that stabilize the cathode‐electrolyte interface, and its formation involves not only the electrochemical process but also the chemical process. Artificially constructed CEI through thermal field induction,^[^ ^76^ ^]^ products generated by chemical reactions,^[^ ^77^ ^]^ additional phases formed by interface modification materials on SE surfaces,^[^ ^78^ ^]^ and interfacial products resulting from high‐temperature sintering of solder^[^ ^79^, ^80^, ^81^ ^]^—all these components that stabilize the interface can be regarded the generalized CEI.

## 3 CEI Diversity in SSLBs with Various SEs

The composition of the CEI is strongly correlated with electrolyte materials. The CEI in SPEs and GPEs still maintain the characteristics of conventional CEI, while inorganic SEs exhibit significant differences, such as S/HEs involve the participation of sulfur/halogen elements in forming a thicker scale CEI layer or CEI‐analogue passivation layer. OEs, depending on the selected interface modification strategy, may form through various pathways including interphases on the SE surface or artificial chemical mixed phases. In addition, when matched with various cathodes, the composition and properties of CEI become even more complex, as the selected cathodes dominate the operating voltage of SSLBs, which directly influences the degree of electrolyte decomposition and the thickness of CEI.

### 3.1 CEI Characteristics Based on Organic SEs

CEIs are dominated by alkyl‐lithium species in SSLBs with organic SEs (GPEs and SPEs), yet the exact secondary composition of the interphase varies with specific electrolyte formulation. GPEs are commonly prepared by through introducing a liquid plasticizer into the polymer backbone. Consequently, their lithium ion transport mechanisms, compared to those of SPEs, more closely resemble those of LEs. Lithium ion transport proceeds through the continuous gel or liquid phase without coupling to polymer‐chain segmental motion in GPEs.^[^ ^82^ ^]^ GPEs are simply divided into two types: solvent‐retaining and polymerized according to its preparation process. In the solvent‐retaining GPEs, a porous matrix commonly employ polymers, such as PEO, polyacrylonitrile (PAN), poly(vinylidene fluoride‐hexafluoro propylene) (PVDF‐HFP), polyimide (PI), poly(urethane) (PU), poly(vinyl chloride) (PVC), polymethylmethacrylate (PMMA), polyvinylidene fluoride (PVDF),^[^ ^83^, ^84^ ^]^ which swells to physically adsorb the liquid plasticizer through capillary and swelling forces, immobilizing the solvent without covalent bonding.^[^ ^85^ ^]^ However, solvent‐retaining GPEs remain vulnerable to leakage under external compression.^[^ ^84^ ^]^ This issue is effectively addressed in polymerized GPEs, which offer a more robust solution. Through thermal or ultraviolet‐initiated polymerization, monomers, plasticizers, and lithium salts are covalently integrated into a 3D polymer network that permanently immobilizes the solvent, eliminating leakage risks.^[^ ^15^, ^86^ ^]^ To date, researchers have successfully investigated the syntheses of tripropylene glycol diacrylate (TPGDA), cyanoethyl polyvinyl alcohol (PVA‐CN), poly(vinyl carbonate) (PVCA), poly(trimethylolpropane triglycidyl ether) (PTTE), poly(1,3‐dioxolane) (poly‐DOL), poly(ethylene glycol) methyl ether acrylate (PEGMEA), and poly(fluoroethylene carbonate) (PFEC)‐based GPEs via polymerization.^[^ ^87^ ^]^

**Table** **1** summarizes CEI constituents and physical parameters observed in the abovementioned two types of GPEs. Because the GPEs is dominated by liquid plasticizers (LE or ionic liquids (IL)),^[^ ^88^ ^]^ the compositions, function, and modification methods of CEI in GPEs‐based SSLBs closely resemble the conventional CEI.^[^ ^89^ ^]^ The CEI compositions are mainly composed of ROCO_2_Li (R represents any alkyl group), LiF, Li_2_CO_3_, Li_x_PO_y_F_z,_ and Li_x_PF_y_, which collectively stabilize the cathode‐electrolyte interface,^[^ ^62^, ^90^, ^91^ ^]^ reduce the interfacial impedance,^[^ ^62^, ^86^ ^]^ buffer the volume change,^[^ ^92^ ^]^ enhance the thermal stability,^[^ ^92^, ^93^ ^]^ and preserve the structural integrity of the cathode materials.^[^ ^87^, ^94^ ^]^ To construct the compatible and stable CEI, the effective strategy of additive regulation has been generally employed to modify the CEI.^[^ ^90^, ^95^ ^]^ In GPEs systems, the additive modifier does not solely target the cathode surface; residual additive molecules also become an integral part of the polymer matrix, altering the intrinsic properties of the electrolyte. For instance, trimethyl phosphate (TMP) retained within PEO‐ or PVDF‐HFP‐based GPEs simultaneously tunes the CEI chemistry and reconstructs the lithium ion solvation sheath via molecular anchoring, thereby accelerating ion transport within the gel.^[^ ^94^ ^]^ Consequently, any additive strategy for CEI optimization in GPEs must be evaluated for its dual impact on both the interphase and the bulk electrolyte.

**Table 1:** Summary of CEI constituents and physical parameters of solvent‐retaining and polymerized GPEs.

GPEs type	Plasticizers	Polymer backbone	Additives	CEI constituents and thickness	Refs.
Solvent‐retaining	1 m LiPF_6_ in DEC/EC/EMC = 2/3/5 wt.%	PE + P‐(VdF‐HFP)	SA + 3 wt.% TMSPB	ROCO_2_Li, LiF, Li_x_PF_y_, Li_x_PO_y_F_z,_ and B─O components (10 nm)	[86]
	1 m LiTFSI in DOL/DME +5 wt.% FEC	PAN/PLLA‐g‐CS electro‐spun membrane	1.25 wt.% LiPAAOB	ROCO_2_Li, LiF, Li_2_CO_3_, Li_2_O, Li_3_N, LiNSO_2_CF_3_ (4 nm)	[62]
	TMP	PVDF‐HFP‐PEO	TMP	LiF, Li_x_PF_y_O_z,_ and organic hybrids (2.7 nm)	[94]
	1 m LiPF_6_ in EC/DMC = 1:1	BTO/PVDF‐HFP nanofiber membrane	BTO	ROCO_2_Li, Li_x_PO_y_F_z_, LiF, and MF_x_ (4.37 nm)	[96]
	1 m LiPF_6_ in EC/DEC/EMC = 3:2:5	PEO + PE + P(VdF‐HFP)	SN	ROCO_2_Li, LiF (20 nm)	[97]
Polymerized	B‐PEGMA + AN	PVC		─C≡N and LiF components (4.7 nm)	[92]
		ipn‐PEA		Amorphous LiF, Li_x_PO_y_F_z_, Li_2_CO_3_ (5 nm)	[90]
	10 m LiTFSI in EC/EMC	P‐DOL	0.05 m LiPF_6_	A fluorine‐rich outer layer and manipulative LiF/organofluorine species (7 nm)	[98]
	0.5 m LiTFSI, 0.5 m LiDFOB, and 5 wt.% LLZTO w. CA+PEGMA	Cellulose membrane		A─C≡N‐rich and LiF‐rich components (7.5 nm)	[91]
	1 m LiPF_6_ in EC/DEC/DMC = 1:1:1	P‐DOL		LiF, Li_x_BO_y_F_z_ (4 nm)	[99]
	SN	P‐DOL		N‐, F‐, and B‐rich inorganic components (7 nm)	[87]
	1 m LiPF_6_ in EC/DMC = 1:1	PEGDA + AN + DEVP + PVDF‐HFP electro‐spun membrane		LiF, Li_2_CO_3_, ROCO_2_Li (3‐6 nm)	[93]
	1 m LiPF_6_ in EC/DEC/DMC	P‐DOL	1 wt.% LiDFOB	Li_x_BO_y_F_z_, LiF, Li_x_PO_y_F_z_ (9 nm)	[95]

To enhance batteries safety, the use of plasticizers in the electrolyte should be avoided to achieve all‐solid‐state batteries. The organic electrolyte without plasticizers are defined as SPEs, which consist of a polymer matrix (e.g., PEO, PAN, PMMA, PVDF, and PVDF‐HFP) swollen with lithium salts, such as LiClO_4_, lithiumbis(trifluoromethanesulfonyl)imide (LiTFSI), lithiumbis(fluorosulfonyl)imide (LiFSI), lithium difluoro(oxalate)borate (LiDFOB), and LiPF_6_. In SPEs, ionic transport relies on the segmental motion of the polymer chains and ion hopping.^[^ ^100^, ^101^, ^102^ ^]^ Additionally, owing to their facile processability, SPEs are often hybridized with ceramic fillers, inorganic electrolytes, or secondary polymers to improve lithium ion transport and mechanical strength of the pristine polymer matrix.^[^ ^83^ ^]^ However, the low intrinsic electrochemical tolerance of SPE has become the most urgent issue in high‐voltage batteries. More importantly, organic SPE‐derived CEIs also exhibit compositional similarity to conventional characteristics, particularly the organic compositions in CEI, such as alkyl lithium compounds, oxidize readily at high‐voltage.^[^ ^31^ ^]^ Therefore, the CEI constituents with high‐voltage stability are essential for SPEs‐based batteries. **Table** **2** shows that the LiDFOB additive and LiF‐rich components are the most prevalent components in high‐voltage polymer‐based SSLBs. This is due to that the borate‐containing component contributes to suppressing the electrochemical oxidation of unstable polymer, while the high‐surface‐energy and chemically robust LiF can yield compact and effective passivation layers to block further SE decomposition.^[^ ^103^ ^]^ Additionally, raising salt concentration to EO:Li⁺ ≤ 6:1 can enhance the oxidative voltage ceiling by forcing nearly complete polymer coordination and contributes to forming the anion‐derived interfacial layers, thus trimming both CEI thickness and detrimental F‐ and S‐rich domains while preserving good compatibility with high‐voltage cathodes.^[^ ^65^ ^]^

**Table 2:** Summary of CEI constituents and physical parameters of SPEs.

Electrolyte composition	Electrode and operation voltage	Additives	CEI constituents and thickness	Refs.
PEO	NCM622‐Li 3–4.2 V	LiDFOB	Li_x_B_x_O_y_ and LiF	[104]
PEO + LiTFSI + LLZTO	NCM622‐Li 2.5–4.3 V	LiPO_2_F_2_, LiFSI, and LiF	ROLi, SO_2_F^−^, Li_x_PO_y_F_z_, and LiF (4.2 nm)	[105]
PEO + LiTFSI	NCM622‐Li 2.8–4.2 V	LiODFB	Li_x_BO_y_F_z_ and LiF‐rich (6–7 nm)	[66]
PEO+LiTFSI (EO:Li = 4:1)	LiCoO_2_‐Li 3.0–4.2/4.4 V	–	Li_2_CO_3_, LiF, C═O, polysulfides (3–6 nm)	[65]
PEO + PVDF + LiTFSI + LLZTO	LiFe_0.5_Mn_0.5_PO_4_‐Li 2.5–4.5 V	LiBODFP and LiF	LiP_x_O_y_F_z_‐rich, LiF, Li_2_CO_3_	[64]
PVDF‐HFP + LiTFSI + LAGP + ionic liquid	NCM811‐Li 3.3–4.5 V	LiDFOB	Li_x_BO_y_F_z_, Li_2_S_2_O_3_, and LiF (3 nm)	[106]

### 3.2 CEI Characteristics Based on Inorganic SEs

Inorganic SEs were classified as either oxides or sulfides. Subsequently, the halide family, such as Li_3_YCl_6_ and Li_3_YBr_6_, were successfully developed, emerging as a new and promising type of inorganic SE.^[^ ^107^, ^108^ ^]^ Unlike rigid oxide electrolytes, both halide and sulfide electrolytes are soft for cold‐pressing, enhancing intimate electrode–cathode contact to eliminate grain‐boundary resistance and enable good lithium ion transport.^[^ ^109^ ^]^ Halide electrolytes can be uniformly represented as Li‐M‐X (M: metal elements; X: halide elements) and some of these species, such as Li_3_InCl_6_, Li_3_YCl_6_, Li_3_ErCl_6_, and Li_3_YBr_6_, have attracted much attention due to their high lithium ionic conductivity and wide electrochemical windows.^[^ ^110^ ^]^ For example, Li_3_InCl_6_ with a layered structure can deliver a lithium ion conductivity close to 2 mS cm^−1^ at room temperature and a stable electrochemical of 4.2 V.^[^ ^111^ ^]^ Among sulfide electrolytes, mainly including thio‐LISICON, Li_10_GeP_2_S_12_‐type (LGPS, derived from thio‐LISICON), and argyrodite,^[^ ^112^ ^]^ argyrodite SEs are the most widely used because they exhibit high ionic conductivity, low cost, and good interfacial compatibility, superior to LGPS and thio‐LISICON‐type electrolytes.^[^ ^109^ ^]^ As typical argyrodite‐type SEs, Li_6_PS_5_X (X = Cl, Br, I), electrolytes deliver good room‐temperature lithium ion conductivity ranging 10^−2^–10^−3^ S cm^−1^ at room temperature,^[^ ^112^ ^]^ which are comparable to that in LEs.

Despite their considerable advantages mentioned above, the electrochemical window of both halide and sulfide electrolytes are insufficient to meet the requirement of high‐voltage batteries. Most sulfides exhibit a narrow electrochemical window, typically only ranging from 1.7–2.3 V.^[^ ^68^, ^113^ ^]^ Similarly, halide SEs like Li_3_InCl_4.8_F_1.2_ undergo electrochemical decomposition at potential over 4 V.^[^ ^12^ ^]^ The above discussions imply that both sulfide and halide electrolytes are intrinsically prone to interfacial side reactions with the electrode during cycling.^[^ ^110^, ^114^ ^]^ Nevertheless, practical SSLBs with sulfide or halide SEs often deliver good electrochemical performance because in situ interfacial interphases and degraded bulk SE act as effective passivation layers, i.e., the generalized CEI we defined above, to suppress continuous interfacial side reactions. The passivating layer constituents of S/HEs are shown in **Table** **3**.

**Table 3:** Summary of CEI‐analogue passivation layer constituents of S/HEs.

Electrolyte type	Electrolyte	Electrode and operation voltage	Passivating layer constituents	Refs.
Sulfide	Li_7_P_3_S_11_	Li_2_RuO_3_‐Li/In 2.0–4.3 V	Phosphates, sulfates, and transition metal sulfides	[13]
	Li_5.5_PS_4.5_Cl_1.5_	NCM85‐Li 2.6–4.5 V	Polysulfides, oxygenated phosphorous, and sulfur species	[57]
	Li_6_PS_5_Cl	LiNbO_3_@S‐NMC811‐Li	Sulfate and phosphate species	[115]
	Li_6_PS_5_Cl	LiCoO_2_‐Li 2.5–4.3 V	Polysulfide (─S─S─), sulfate (SO_4_^2−^), and sulfite (SO_3_^2−^),	[116]
Halide	Li_2_YCl_2.5_Br_1.5_O_0.5_	NCM83‐Li/In 3.0–4.3 V	YOCl and Y_2_O_3_	[117]
	Li_2.5_ZrCl_5_F_0.5_O_0.5_	NCM955‐Li 2.5–4.35 V	F‐rich species	[118]
	Li_3_InCl_4.8_F_1.2_	LiCoO_2_‐In 2.6–4.47 V	LiF and F‐rich species	[71]

Unlike H/SEs, OEs do not face a serious challenge with electrochemical stability. In such SSLBs with typical OEs, such as garnet‐type (e.g., Li_7_La_3_Zr_2_O_12_, LLZO), sodium superionic conductor‐type (e.g., Li_1+x_Al_x_Ti_2‐x_(PO_4_)_3_, LATP), and perovskite‐type (e.g., Li_3x_La_2/3‐x_TiO_3_, LLTO),^[^ ^119^ ^]^ the rigid OEs form solid‐solid contact with electrodes that severely hinder electrochemical performance. Thus, to ensure intimate interfacial contact and suppress impedance, the effective strategies are to introduce liquid or flexible electrolytes (such as LE, GPEs, and SPEs) or other soft interphases. These cathode‐side interfacial modifications share similar properties and mechanisms with CEI described in the previous organic electrolytes. Notably, benefiting from the high thermal and chemical stability of OEs, a series of interfacial engineering strategies, such as magnetron sputtering,^[^ ^120^, ^121^ ^]^ and cathode‐electrolyte co‐sintering^[^ ^79^, ^80^, ^81^ ^]^—are readily accessible. These exclusive treatments endow OE‐based SSLBs a CEI whose composition and characteristics diverge markedly from those of other SSLB systems. In thin‐film SSLBs based on magnetron sputtering, the absence of conductive carbon and polymeric binders in the cathode further reshapes the CEI chemistry. Owing to the relatively simple phase composition and the absence of complex side reactions in the cathode, the requirement for the CEI to accommodate volume fluctuations are significantly diminished. The accompanying high energy of the magnetron sputtering process enables the solid‐state battery components to react even without electrochemical reactions, leading to the formation of the chemical CEI. During the co‐sintering process between cathode and OEs, the high‐temperature field leads to the formation of chemical CEI, whose characteristics and compositions are profoundly depended on the properties of the cathodes, solder, additives, and the inherent features of OEs. The resulting highly integrated hybrid interphase, formed under the co‐sintering process, contributes to intimate physical contact and high thermodynamic stability while enhances the complexity and diversity of CEI composition. Critically, subsequent electrochemical cycling further alters CEI characteristics as well, deserving equal attentions.

## 4 CEI Regulation Strategies in SSLBs

**Figure 3:**
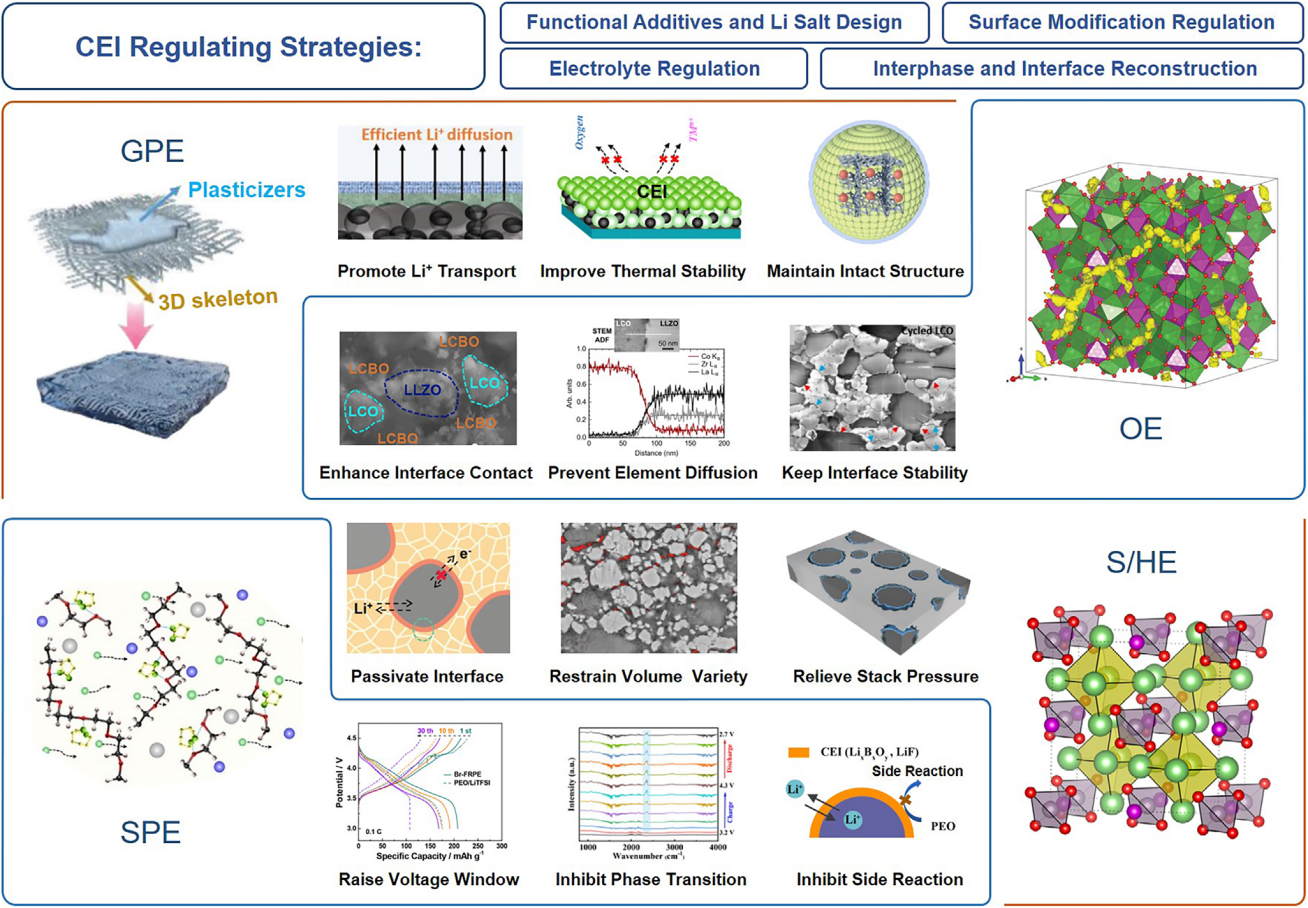
Various CEI regulating strategies for stabilizing interfaces in different solid‐state electrolyte‐based lithium batteries.^[^ ^13^, ^35^, ^55^, ^62^, ^67^, ^79^, ^81^, ^86^, ^91^, ^104^, ^122^, ^123^, ^124^, ^125^, ^126^ ^]^ Copyright 2022, American Chemical Society. Copyright 2021, Elsevier. Copyright 2021, Elsevier. Copyright 2023, Wiley‐VCH. Copyright 2018, Elsevier. Copyright 2018, American Chemical Society. Copyright 2023, American Chemical Society. Copyright 2023, Wiley‐VCH. Copyright 2023, Wiley‐VCH. Copyright 2019, Royal Society of Chemistry. Copyright 2023, Wiley‐VCH. Copyright 2021, Wiley‐VCH.

Owing to diverse matching combinations between cathode materials and SEs, the functional requirements for CEI vary significantly across different SSLBs. To address these varying demands, general strategies for CEI regulation, including surface modification, functional additive design, and electrolyte engineering, have been developed for various types of SEs, such as GPEs, SPEs, SEs, HEs, and OEs. Effective modulation of the CEI enables multiple performance improvements, such as preserving structural integrity,^[^ ^86^ ^]^ improving thermal stability,^[^ ^35^ ^]^ facilitating lithium ion transport,^[^ ^91^ ^]^ enhancing interfacial contact,^[^ ^79^ ^]^ suppressing element interdiffusion,^[^ ^122^ ^]^ maintaining interface stability,^[^ ^81^ ^]^ inhibiting side reaction,^[^ ^104^ ^]^ inhibiting phase transition,^[^ ^123^ ^]^ raising voltage window,^[^ ^124^ ^]^ relieving stack pressure,^[^ ^55^ ^]^ restraining volume variety,^[^ ^55^ ^]^ and passivating interface (**Figure** **3**).^[^ ^13^ ^]^

### 4.1 Surface Modification Regulation

**Figure 4:**
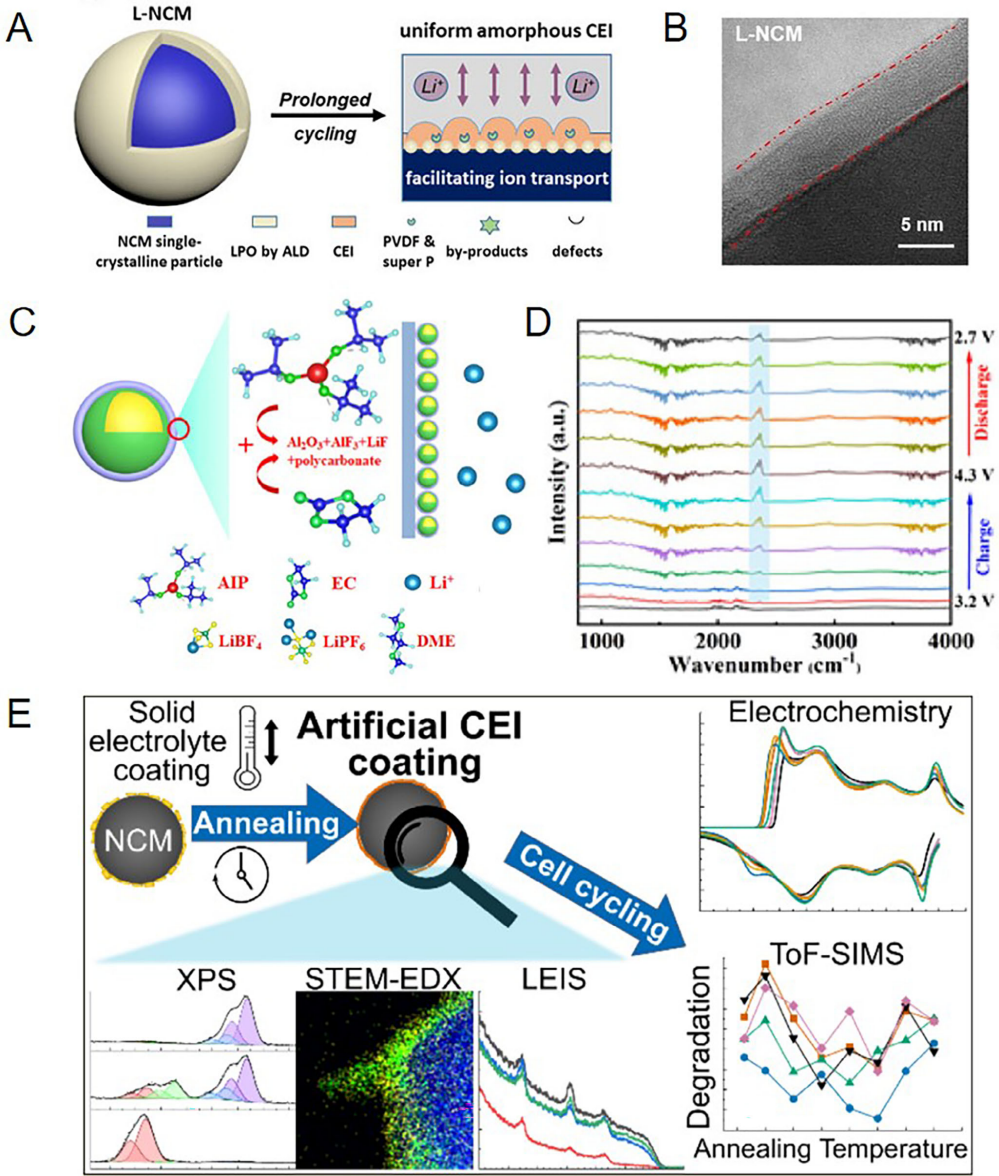
A) Mechanism schematic of the surface degradation and regulating interface in L‐NCM|interpenetrating network of poly(etheracrylate) (ipn‐PEA) (electrode/electrolyte) batteries during charging and discharging process. B) The TEM images of the LiPO_3_‐coated L‐NCM.^[^ ^90^ ^]^ Copyright 2022, Wiley‐VCH. C) Schematic illustration of the CEI layers formation mechanism for the cathode. D) FT‐IR spectral and the corresponding contour maps of the cathode/electrolyte interface at different charging and discharging process.^[^ ^123^ ^]^ Copyright 2023, Elsevier. E) Schematic of artificial CEI coating after the oxidative annealing process in LPSCl‐based ASSLBs.^[^ ^131^ ^]^ Copyright 2025, American Chemical Society.

Surface coating was initially employed to isolate the SEs from the cathode materials.^[^ ^127^ ^]^ A coating layer with low reactivity helps enhance the stability of the cathode interface. However, inhomogeneous coating layers often contain defects, which compromise complete interface passivation.^[^ ^72^, ^128^, ^129^ ^]^ Therefore, achieving a truly stable interface ultimately depends on the formation of a well‐designed CEI and CEI‐analogue passivation layers. For instance, Guo et al. applied the Li_3_PO_4_ (LPO) coating layer on Ni‐rich cathodes via atomic layer deposition. In situ atomic force microscopy revealed that surface defects on pristine LiNi_x_Co_1‐x‐y_Mn_y_O_2_ (NCM) particles deteriorate during electrochemical cycling in GPE‐based batteries, resulting in inhomogeneous CEI and unstable decomposition products. The LPO coating could effectively control the content of amorphous LiF and other by‐products, contributing to forming uniform and thin CEI without surface defects (**Figure** **4**A,B).^[^ ^90^ ^]^ Moreover, surface coatings could regulate CEI compositions via changing surface properties of cathode materials, such as reducing surface reactivity, lowering interfacial impedance, and elevating lithium ion transport kinetics. Liu et al. utilized isopropanol Al (AIP) to coat NMC811.^[^ ^123^ ^]^ The strong nucleophilic RO^−^ groups in AIP induced the polymerization of EC, forming a CEI layer with high antioxidant compositions of Al_2_O_3_, AlF_3_, and polycarbonate, which significantly improved the mechanical strength of the CEI layer (Figure 4C,D). The dense and inorganic‐rich CEI not only improved the interfacial stability and inhibited the irreversible phase transitions in the NCM, but also facilitated the diffusion of lithium ion between NCM particles and alleviated their mechanical cracking over prolonged cycling.^[^ ^130^ ^]^

In SSLBs employing electrolytes of limited electrochemical stability, interfacial stability at the cathode side is predominantly governed by on the CEI. The electrochemical activity of the cathode surface plays a decisive role in determining the formation quality and passivation capability of CEI. As demonstrated by Qiu et al., PEO can form a stable interface with LiFePO_4_ (LFP) cathode while undergoes severe side reaction with LCO, even though both systems operate above the electrochemical stability window of PEO.^[^ ^72^ ^]^ This difference arises because the decomposition products on LFP possess sufficient lithium ion conductivity and unexpectedly facilitate a relatively stable CEI between the cathode and PEO. In contrast, the highly oxidizing surface of delithiated LCO at high‐voltage catalytically accelerates PEO decomposition, resulting in uncontrolled CEI growth and rapid performance degradation. Notably, when LCO is well coated with Li_1.4_Al_0.4_Ti_1.6_(PO_4_)_3_, the modified cathode exhibits reduced surface reactivity, promoting the formation of a stable CEI and thus delivering significantly improved electrochemical performance with 88.6% capacity retention after 50 cycles.

These results underscore that, for oxidation‐sensitive solid electrolytes, the properties of interfacial decomposition products determine overall battery performance. And the surface coating layer can effectively regulate the CEI components and enhance interfacial stability. Similarly, Kissel et al. also reported the polycrystalline LiNi_0.85_Co_0.10_Mn_0.05_O_2_ (NCM85) cathodes pre‐coated with Li_3_PS_4_ precursor before an oxidative annealing process in Li_6_PS_5_Cl (LPSCl)‐based all‐solid‐state lithium batteries (ASSLBs) (Figure 4E).^[^ ^131^ ^]^ This treatment resulted in a protective surface layer containing Li_2_SO_4_, Li_2_CO_3_, and LiOH, which considerably mitigated interfacial side reactions. The modified interface exhibited reduced relaxation potentials, lower polarization, and fewer oxygenated sulfur/phosphorus species in degradation products. Consequently, the batteries with coated NCM85 exhibit higher initial Coulomb efficiency and enhanced cycling performance.

### 4.2 Design of Functional Additives and Lithium Salts

**Figure 5:**
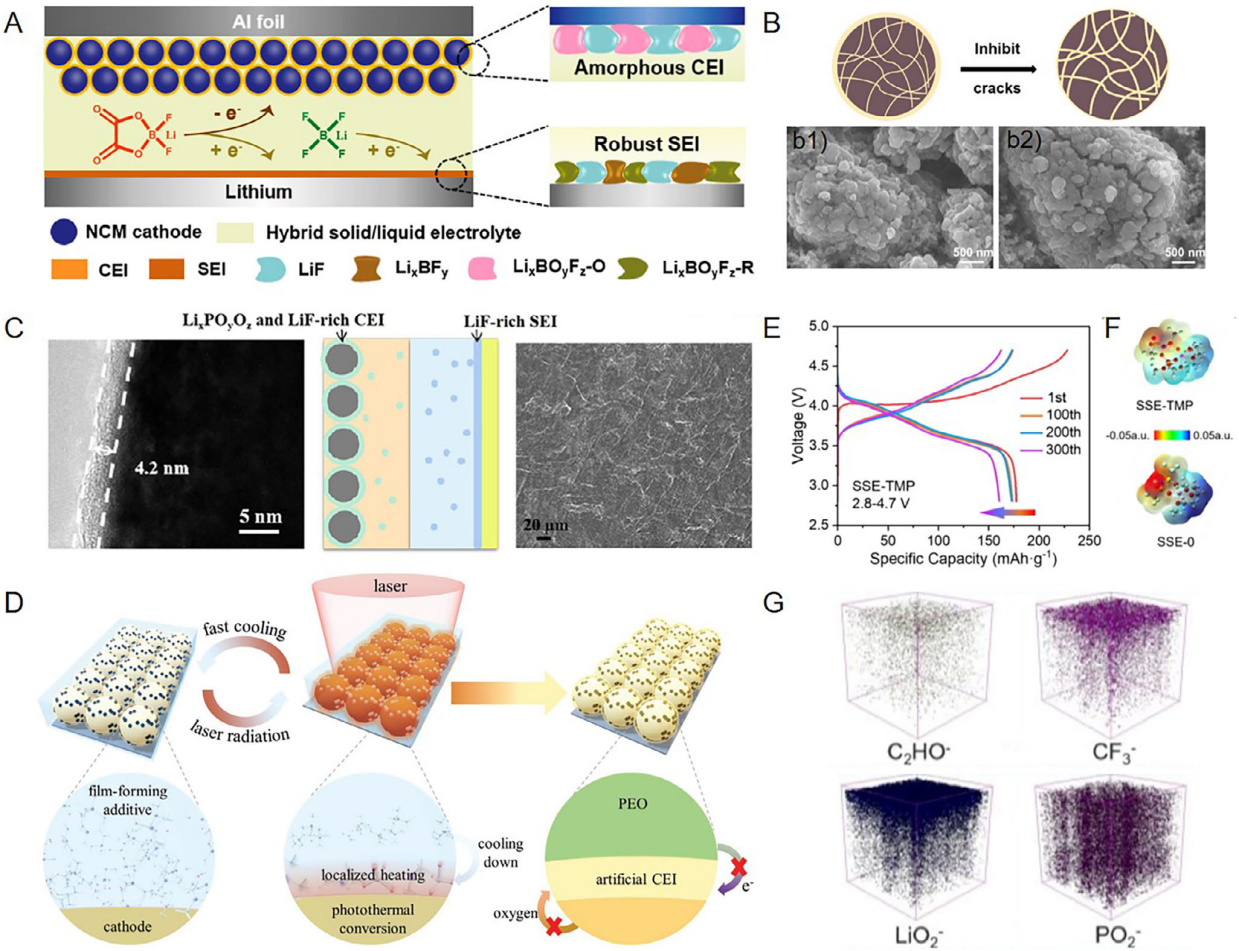
A) Schematic illustration of the detailed chemical constituents of amorphous CEI in quasi‐solid‐state Li‐metal batteries. B) Schematic illustration of the polycrystal NCM622 cathode during cycling. SEM images of the NCM622 composite cathode in B1) dual interface amorphous CEI/SEI protection and B2) the LE‐based batteries after 100 cycles.^[^ ^99^ ^]^ Copyright 2021, American Chemical Society. C) the TEM image of cycled cathode, schematic illustration of CEI generation mechanism, and SEM image of Li anode from cycled symmetric batteries with anode‐side solid electrolyte‐10LiFSI, respectively.^[^ ^105^ ^]^ Copyright 2023, American Chemical Society. D) Schematic illustration of the CEI generation process induced by pulsed laser irradiation.^[^ ^76^ ^]^ Copyright 2022, Wiley‐VCH. E) Galvanostatic charge and discharge curves for different cycles based on SE‐TMP during 2.8–4.7 V. F) Electrostatic potential mapping for two different solvated molecules. G) 3D TOF‐SIMS reconstruction of SE‐TMP‐based NCM811 after the 300 cycles.^[^ ^94^ ^]^ Copyright 2023, Elsevier.

The incorporation of functional additives is one of the most convenient and effective strategies to tailor the composition and properties of the CEI, thereby improving the cathode‐electrolyte interfacial compatibility.^[^ ^30^ ^]^ Boron‐containing additives, such as lithium bis(oxalato)borate (LiBOB) and LiDFOB, are widely recognized for enhancing the high‐voltage electrochemical stability of SSLBs.^[^ ^132^ ^]^ Liang et al. have developed an amorphous CEI with highly plastic via in situ synergistic conversion of LiDFOB and LiBF_4_ in a hybrid solid‐liquid electrolyte system polydioxolane‐ethylene carbonate/dimethyl carbonate/diethyl carbonate (EC/DMC/DEC). This approach reconciles multiple interfacial compatibilities and enables excellent cycling stability even under harsh conditions, including high cathode loading, high‐voltage (4.5 V), and high‐temperature (45 °C) (**Figure** **5**A,B).^[^ ^99^ ^]^ Specifically, the LiBF_4_ salt slows the rapid consumption of LiDFOB at the cathode, resulting in a CEI with distinct chemical composition and inorganic content. This robust interphase functioned as a dynamic protective layer, maintaining stability at 4.5 V. It also effectively accommodates lattice strain and mitigates structural collapse of the cathode during long‐term cycling, significantly extending the life of the pouch cell. In addition, fluorine‐rich species are considered highly beneficial for CEI formation due to their high surface energy and superior resistance against HF corrosion.^[^ ^103^ ^]^ Liu et al. added LiPO_2_F_2_ and LiFSI into PEO to regulate the CEI on the NMC622 surface (Figure 5C).^[^ ^105^ ^]^ LiPO_2_F_2_ promoted a robust and uniform CEI enriched with Li_x_PO_y_F_z_ and LiF, improving the stability of SPE at high oxidation voltage.

Conventional CEI formation through electrochemical reactions with additives offers limited controllability in thickness and morphology. The functional CEI components derived from the additive decomposition often intermix with electrolyte breakdown products, thus reducing the utilization of additives. Moreover, the additives that improve the quality of CEI may adversely affect the anode SEI. To achieve precise control over CEI properties, Tang et al. utilized a laser beam technique to construct a continuous and multi‐scale artificial CEI.^[^ ^76^ ^]^ This technique enables selective decomposition of precursor salts to tailor CEI composition and structure according to the desired CEI integrities. They studied the laser‐induced decomposition of various additives, including tris(trimethylsilyl)phosphate (TMSP), 1,3‐propane sultone (PS), tris(trimethylsilyl)borate (TMSB), and LiDFOB. As a result, the laser‐induced NMC811 cathode can achieve stable cycling at 4.3 V up to 2 C, alongside a 43.2% decrease in leakage current, even when directly assembled with pristine PEO. Supported by high temperature, in situ phase tracking, and differential scanning calorimetry, the laser‐processed electrode additionally exhibited mitigated layered‐to‐spinel phase transition during long‐term cycling, as well as a 75.5% reduction in self‐discharge at 55 °C. This observation is consistent with the suppression of PEO oxidation and the alleviation of oxygen release under elevated temperature conditions. (Figure 5D).

**Figure 6:**
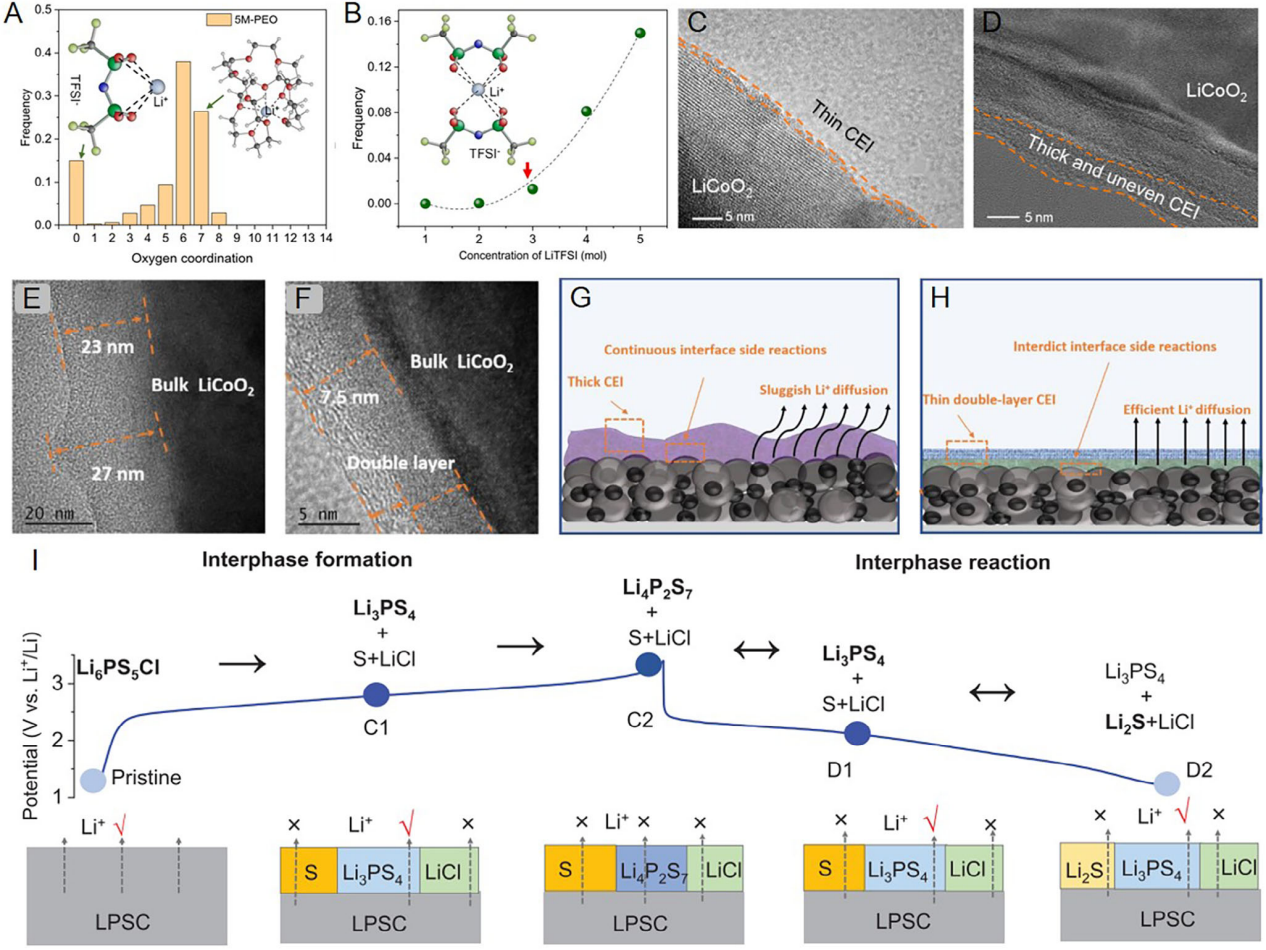
A) Coordination distribution with O from EO in 5 m PEO solid electrolytes. B) Frequency of lithium ion coordination without O from EO with different LiTFSI concentration. High‐resolution TEM images of the ASSLBs using C) PEO(16:1)/PEO(4:1) and D) PEO(16:1) solid electrolytes after 20 cycles.^[^ ^65^ ^]^ Copyright 2023, Elsevier. E,F) Typical TEM image of CEI layers of random areas on the surface of LCO particles with E) PGL and F) cyano reinforced high‐voltage EO‐based SPE (CA‐PGL) after 50 cycles at 4.4 V and 60 °C. G, H) Schematic illustration of G) the thick CEI formed in PGL and H) the thin double‐layer CEI formed in CA‐PGL.^[^ ^91^ ^]^ Copyright 2021, Elsevier. I) The stages of interphase formation and interphase reaction featuring the redox products at each representative state, with the upper panel showing the voltage profile and the bottom showing the distribution of products and the capability for lithium ion flow at the interphase.^[^ ^139^ ^]^ Copyright 2026, Wiley‐VCH.

Beyond the chemical nature of electrolyte components like additives and lithium salts, their concentration also plays a critical role in determining the properties and functions of CEI. High concentration of Li salt has been proved to effectively extend the electrochemical stability window and increase the oxidation potential of conventional LEs.^[^ ^133^ ^]^ For example, Hou et al. used a highly concentrated electrolyte with 10 m LiTFSI and a small amount of LiPF_6_ to modulate the proportion of F‐S organofluoride and inorganic LiF in the CEI. The enriched anions undergo preferential oxidation, facilitating the formation of a F‐rich and dense CEI.^[^ ^98^ ^]^ Similarly, a localized high concentration of Li environment was constructed by adding TMP liquid additives to the polymer electrolyte. These additives induced the in situ formation of CEI enriched with LiF, Li_x_PF_y_O_z_, and organic mixtures, a process achieved via TMP molecular reconstruction of the lithium ion solvation structure, thereby stabilizing the structural integrity of the nickel‐rich cathode at 4.7 V (Figure 5E–G).^[^ ^94^ ^]^ Moreover, high concentration of Li salt can also modify the lithium ion solvation structure without compromising ionic conductivity or bulk material properties.^[^ ^134^ ^]^ Xiong et al. demonstrated that PEO‐based SPE with high lithium ion concentration (EO:Li^+^ = 4:1) possesses a high oxidation potential (5.19 V) because most of the EOs participated in coordinated solvation structures,^[^ ^65^ ^]^ thereby reducing the polymer reactivity.^[^ ^135^ ^]^ The high lithium ion‐concentration PEO inhibits the interfacial degradation kinetics, eliminates the electronic conduction in CEI, and inhibits the irreversible phase transition from LCO to Co_3_O_4_. In contrast, a low‐concentration PEO electrolyte (EO:Li^+^ = 16:1) leads to the formation of S‐rich CEI, which is ≈10 nm thick and electronically conductive, on the LCO surface, compromising the electrochemical stability. The CEI formed on LCO with high‐concentration PEO is only 3–6 nm and has no S‐containing species (**Figure** **6**A–D). Wang et al. further designed a CEI composition specifically suited for the SPE‐based batteries through using the polymer‐in‐salt electrolyte. The polymer‐in‐salt electrolyte facilitates the formation of a CEI with an organic derivative on the surface and dense LiF in the inner layer. This CEI inhibits the diffusion of TM and provides solid‐phase protection against the continuous decomposition of ether‐based SPEs. The optimized NCM cathode‐based ASSLB exhibits an excellent capacity of 141.4 mAh g^−1^ at 1 C with capacity retention of 81.6% after 400 cycles.^[^ ^136^ ^]^

Furthermore, CEI regulation should not focus exclusively on additives. The role of fillers in the electrolyte should not be overlooked. It has been found that the BaTiO_3_‐Li_0.33_La_0.56_TiO_3‐x_ nanowires can tightly anchor N, N‐dimethylformamide and restrict its side reactions with NCM811, which greatly weakens the space charge layer at the cathode‐electrolyte interface, exhibiting decreased polarization from 494 to 179 mV.^[^ ^137^ ^]^ Additionally, Tian et al. have reported that incorporating a Cu‐metal–organic framework (MOF) filler into PEO can strongly anchor TFSI^−^ anion.^[^ ^138^ ^]^ This promotes the formation of inorganic‐rich (NaF and Na_3_N) CEI and SEI, enhancing CEI/SEI interfacial stability. Consequently, Na‐ion full cells demonstrate outstanding cyclability, maintaining over 2000 cycles.

### 4.3 Electrolyte Bulk Phase Regulation

The components of CEI are also largely derived from the electrolyte decomposition products, indicating that the electrolyte matrix directly influences CEI properties, and its regulation becomes a primary design lever. In GPE‐based SSLBs, the CEI formation is not only plasticizers and additives but also the polymer backbone. For instance, Lv et al. engineered a 2‐cyanoethyl acrylate (CA) reinforced poly (ethylene glycol) methyl ether acrylate via in situ polymerization, wherein the ─C≡N with low negative electrostatic potential enable the CEI enriched in ─C≡N and LiF (Figure 6E–H). This design further enhances the compatibility between EO‐based SPE and LCO cathode, inhibiting the decomposition of ether functional groups. Consequently, LCO‐based SPE batteries exhibit excellent cyclability for 500 cycles under conditions of 4.4 V and 60 °C.^[^ ^91^ ^]^ Furthermore, combining electrolyte and additive design amplifies these benefits and enhances interfacial stability. Liu reported a LiDFOB and LiTFSI enhanced DOL electrolyte with succinonitrile (SN). Within this GPE, electrolytes matrix and additives jointly form an N, F, B‐rich CEIs, which can suppress unwanted side reaction, reduce the energy barrier for lithium ion surface transport, and inhibit TM dissolution.^[^ ^87^ ^]^ In addition to the functional groups of the electrolyte, its derived products also have a significant impact on the formation of CEI. Zheng et al. found that the PEO‐derived radical facilitated the reconstruction of a chemo‐mechanically stable and lithium ion‐conductive CEI layer, which comprised abundant inorganic components (LiF, Li_3_PO_4_, and Li_x_PO_y_F_z_) distributed within lithiated organic macromolecules. Notably, this in situ formed CEI exerts an effective passivation effect on the catalytic active sites of LCO, offering direct protection against PEO. As a result, the layered structure of LCO remains well‐preserved during high‐voltage cycling. Following 1000 cycles, the assembled batteries maintain roughly 84% of its capacity at 1 C at 60 °C.

Inorganic SEs focus on the material modification to optimize CEI. Shen et al. developed an of F^−^/O^2−^ anion‐engineered halide superionic conductors (Li_2.5_ZrCl_5_F_0.5_O_0.5_, LZCFO) with enhanced stability under high‐voltage.^[^ ^118^ ^]^ The introduction of F^−^ enhances the thermodynamic stability of LZCFO and promotes a robust CEI, while O^2−^ increases lithium ion transport within the anionic framework. This modified electrolyte enabled a highly uniform CEI with 4 nm and enriched with F‐containing species, effectively protecting the cathodes from serious side reactions. The introduction of highly amorphous species with disordered localized structures brings a novel lithium ion transport mechanism distinct from crystalline state processes, mitigating the negative impact of F^−^ on ionic conductivity. Consequently, an ASSLB constructed with NCM955 and LZCFO achieves a substantial capacity of 207.1 mAh g^−1^ within a voltage range of 2.5–4.35 V vs Li/Li^+^, along with impressive cycling stability, retaining 81.2% of its capacity after 500 cycles at a charge rate of 0.5 C. Regulating the interphase redox reversibility of the sulfide catholyte could directly affect CEI. Shen et al. validated it through a novel sulfide electrolyte of Li_6+x_P_1−x_W_x_S_5_I (LPWSI).^[^ ^139^ ^]^ The characterizations confirm that WS_2_ mixed ionic‐electronic conductors enhance the interphase reaction from Li_4_P_2_S_7_ to Li_3_PS_4_, preventing irreversible accumulation of resistive P_2_S_7_^4−^ species and elevating the interphase stability of catholyte. Using this LPWSI catholyte, the ambient‐temperature ASSLB maintains 92.2% capacity over 400 cycles at 0.2 C, starting with an areal capacity of 1.95 mA h cm^−2^. Additionally, the cells show excellent stability over 1000 cycles at 1 and 2 C rates. (Figure 6I).

### 4.4 Interphase and Interface Reconstruction

**Figure 7:**
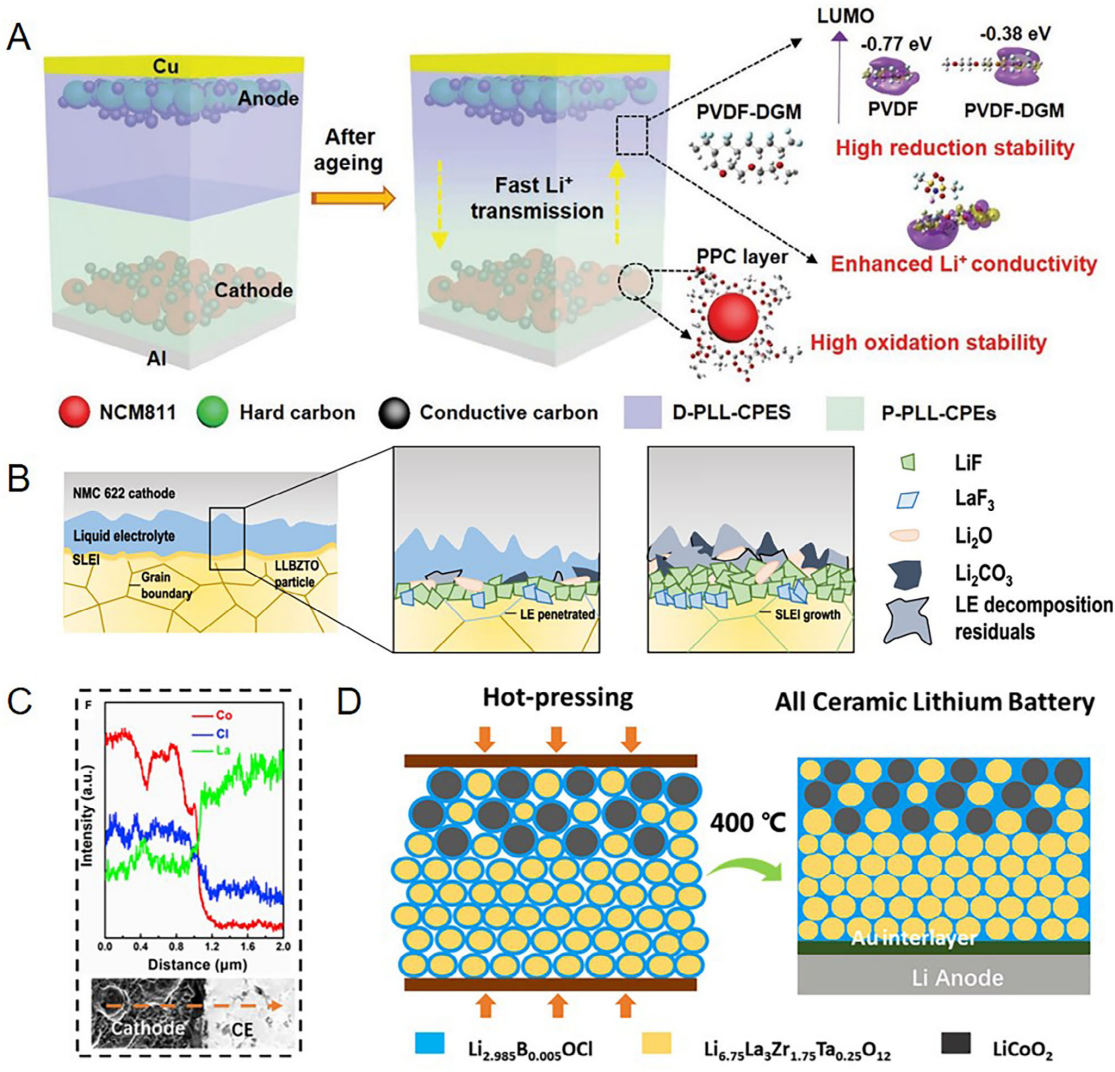
A) Schematic illustration of the design strategy of quasi‐double‐layer CPEs and polymer solid‐state batteries.^[^ ^140^ ^]^ Copyright 2021, Wiley‐VCH. B) Schematic representation of the formation and expansion of SLEI.^[^ ^141^ ^]^ Copyright 2022, American Chemical Society. C) Line‐scanning analysis of the cathode/electrolyte interfaces after hot pressing. D) Schematic illustration of Li_2.985_B_0.005_OCl surface coating and all‐ceramic lithium battery assembling under hot pressing.^[^ ^80^ ^]^ Copyright 2020, Elsevier.

Achieving interfacial compatibility between SEs and high‐voltage cathodes remains a critical challenge due to electrochemical instability and mechanical mismatch. Interfacial reconstruction has emerged as an effective strategy to addresses this issue by constructing artificial interphases that fundamentally modify cathode‐electrolyte interactions. This approach either replaces unstable native CEI or chemically modifies their composition to establish electrochemically robust interfaces. For instance, Pan et al. synthesized an asymmetric composite electrolytes of PVDF/LLZO/LiTFSI, where the cathode interface employed propylene carbonate (PC) monomers for in situ polymerization and constructed an artificial CEI‐analogue layer with 15–30 nm, enhancing the oxidative stability of composite polymer electrolytes (CPEs) at the cathode side. Finally, NCM811‐based CPEs Li cell delivers 136.5 mAh g^−1^ at 1 C during 3–4.8 V (**Figure** **7**A).^[^ ^140^ ^]^ Cho et al. demonstrated a multifunctional interphase material consisting of a pyrrolidinium‐based ionic liquid and a polyethylene oxide polymer with lithium salt, which relieved the interfacial compatibility between LGPS and NCM811 cathode.^[^ ^140^ ^]^ This engineered interphase enables NCM811|LGPS electrode to deliver a capacity of 166 mAh g^−1^ at 25 °C in batteries.

A trace of LEs is routinely introduced to wet the cathode‐electrolyte interface and enhance interface contact for OEs‐based SSLBs. Therefore, it is important to consider the compatibility between the LE and the oxide electrolyte. Notably, the solid‐liquid electrolyte interphase (SLEI) also affects interfacial impedance and lithium ion transport for batteries, it conforms to the generalized CEI and collaboratively stabilizes the interface with the CEI, influencing the morphology and structural stability of the electrode.^[^ ^78^ ^]^ Yan et al. found the failure of OEs‐based SSLB with LE modification might be attributed to the exhaustion of LE at the interface. The SLEI, transformed from the dried LE, exhibited an uneven thickness and thus failed to inhibit LE decomposition. The optimized SLEI, composed of LiF, LaF_3_, Li_2_O, and Li_2_CO_3_, was formed on the surface of Li_6.5_La_2.9_Ba_0.1_Zr_1.4_Ta_0.6_O_12_ (LLBZTO). A thicker SLEI, in conjunction with CEIs generated by conventional ester LE, enables better interface compatibility and long‐term batteries cycling (Figure 7B).^[^ ^141^ ^]^ Additionally, OEs‐based batteries can utilize modification strategies similar to those in liquid LIBs to optimize interphase, such as introducing film‐formation additives or incorporating high‐concentration lithium salts. Sarkar et al. proposed a strategy employing AlCl_3_ as a Lewis acid together with FEC for interface modification.^[^ ^142^ ^]^ This devised strategy aims to suppress side reactions between LE and LLZO by fabricating a mechanically robust and ionic conduction Al‐rich interphase on LLZO. Furthermore, it promotes the formation of a CEIs containing Al_2_O_3_ on the surface, which effectively scavenge HF.

Owing to the excellent thermal stability of the OEs, researchers have developed a stable cathode‐electrolyte interface by applying co‐sintering technology. Under high‐temperature thermal fields, a chemically stabilized interphase is preconstructed between cathodes and electrolytes with the assistance of solder, concurrently establishing intimate physical contact at interfaces. Feng et al. developed the ternary doped solder material Li_2.985_B_0.005_OCl as an artificial interphase, which chemically stabilizes the LCO and LLZO interface while inhibiting La and Co interdiffusion (Figure 7C,D).^[^ ^80^ ^]^ Meanwhile, this ternary doped solder enables rapid lithium ion transport, maintains low cathode/electrolyte interfacial resistance (386 Ω cm^−2^), and mitigates stress/strain damage of batteries via elastic modulus during cycling.

### 4.5 Industrial Viability of Regulation Strategies

The diversity of CEI regulation strategies has been summarized in detail, and to gain more insight, their scalability, cost, and compatibility with industrial manufacturing also merit attention. The surface modification is an effective strategy to change surface properties of cathode materials, its industrial viability depends on the specific technology routes, such as sol–gel, mechanical blending, and atom layer deposition (ALD). Both the sol–gel and mechanical blending methods are highly scalable. Mechanical blending is a low‐cost option with high production compatibility, as it utilizes traditional mixing equipment. Although its coating precision is limited, it is still widely used in industrial production. Conversely, the sol–gel process incurs higher costs, primarily driven by the need for specialized equipment, liquid‐phase solvents, and additional sintering steps. It can serve as an important supplementary technology in industry. However, precise coating technologies like ALD are hindered by extremely high costs and insufficient scalability, making them hard to be effectively applied in industry.

Currently, the most widely used and cost‐effective CEI regulation strategy is to add a small amount of functional additives to the electrolyte. The high scalability of this approach stems from the versatile application of additives across various electrolyte systems and electrode materials. The cost is extremely low, as only a small amount (such as <5 wt.%) is typically required to deliver a substantial performance improvement. Moreover, it offers exceptional compatibility with existing industrial processes because the introduction of additives only requires simple mixing during the electrolyte formulation stage without any significant retrofitting of current production infrastructure. It should be noted that the conventional liquid‐phase additives are difficult to be applied in inorganic SEs, and the solid‐phase additives often face issues with utilization efficiency, potentially increasing costs. Conversely, super concentrated organic SEs represent an important trend, capable of forming more stable and inorganic‐rich CEI. This approach exhibits commendable industrial compatibility similar to the introduction of additives for organic SEs. However, it comes with drawbacks that must be mitigated in manufacturing. The high lithium salt required for high‐concentration electrolytes leads to substantial costs, and the elevated viscosity of these electrolytes can result in poor electrode wettability, posing challenges for injection efficiency and battery performance.

Electrolyte bulk‐phase regulation is a highly scalable strategy for tailoring the CEI, as it is directly integrated into electrolyte material production. For organic SEs, this is achieved through the introduction of functional groups or formulation design for the electrolyte. The former often increases costs and reduces compatibility due to extra processing steps, whereas formulation design is cost‐effective, depending on material selection. In contrast, for inorganic SEs, elemental doping serves as the primary method of electrolyte regulation, and it generally incurs minimal additional costs. Crucially, both formulation design and elemental doping require no extraneous steps, granting them superior industrial compatibility.

Utilizing interphase reconstruction to ensure the properties of the CEI are largely decoupled from the bulk electrolyte. This approach encompasses organic strategies, such as in situ interfacial polymerization and liquid electrolyte wetting, and inorganic approaches like co‐sintering. Among these, interface electrolyte wetting possesses high scalability, low cost, and strong industrial compatibility because it relies entirely on the intrinsic properties of the liquid‐electrolyte. In situ interfacial polymerization introduces an additional heat‐induced polymerization step to the baseline process, which moderately increases cost and slightly compromises production compatibility. Meanwhile, the co‐sintering method faces scalability challenges, mainly due to the need for precise control over temperature and the sintering environment. The heating process often entails higher expenses due to the requirement for high‐temperature furnaces or other specialized equipment, resulting in relatively low compatibility with conventional battery manufacturing lines.

Overall, organic strategies demonstrate greater potential in scalability and industrial compatibility, with costs remaining relatively manageable. In contrast, although inorganic strategies can effectively improve interfacial stability, their industrial adoption will require further resolution of cost‐ and compatibility‐related challenges.

## 5 CEI for Addressing Challenges in SSLBs

### 5.1 Low Mobility of SEs and Physical Contact Loss

**Figure 8:**
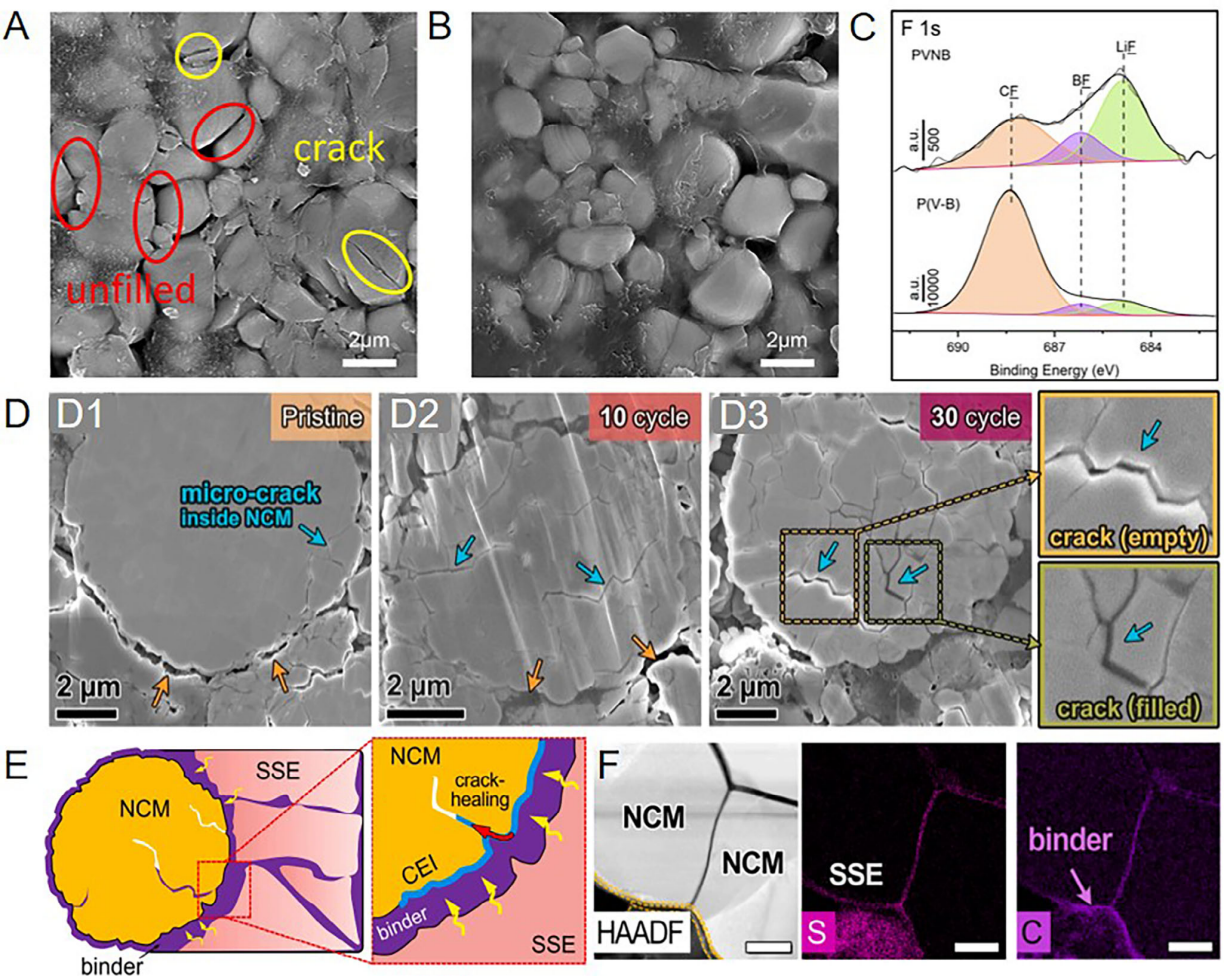
SEM images of the NCM electrode surface in A) poly(ethylene glycol) methacrylate containing cyclic boroxine groups into poly(vinylene carbonate) (P(V‐B)) and B) organoboron‐ and cyano‐grafted polymer electrolyte (PVNB) electrolytes after 50 cycles. C) F 1s XPS spectra of the NCM cathodes of the Li|PVNB|NCM811 cells after 50 cycles.^[^ ^92^ ^]^ Copyright 2023, American Chemical Society. D) Microcracks (shown as blue arrows) observed in secondary NCM particle (pristine and after different cycles) under stack pressure. The magnified regions indicate empty (yellow box) and filled cracks (green box), respectively. E) Schematic diagram of the microcrack self‐healing process. F) SEM and energy dispersive spectrometer elements distribution near the local NCM region of the sample subjected to 30 cycles. The crack‐healing process, where cracks are filled with S and C elements, is observed for the microcrack.^[^ ^145^ ^]^ Copyright 2023, Elsevier.

After formation, the CEI is not static. During electrochemical processes, the CEI undergoes volume changes and even break.^[^ ^143^ ^]^ In LE‐based LIBs, liquid solvent immediately infiltrates these cracks, sustaining fresh reactions and continuously regenerating the CEI.^[^ ^144^ ^]^ However, due to the poor mobility of SEs, it is difficult to repair localized physical pores and newly form complete CEI in SSLBs, driving impedance rise and interfacial detachment. Therefore, it is critical that the CEI in SSLBs withstand volume changes and maintain continuous lithium ion transport within the composite cathode.^[^ ^62^, ^99^ ^]^ To achieve this functionality, Liu et al. demonstrated that organic‐rich CEIs are unable to accommodate large volume changes.^[^ ^92^ ^]^ Thus, they proposed the organoboron‐ and cyano‐grafted solid polymer electrolyte capable of forming an organic–inorganic composite CEI. In such CEI, the C≡N‐rich constituents could anchor firmly with the cathode surface to maintain intimate contact and provide lithium ion transport channels, as well as the LiF‐rich constituents contribute to releasing stress caused by the change of electrode volume via weak LiF–cathode bonding (**Figure** **8**A–C).^[^ ^42^ ^]^ In SSLBs with inorganic SEs, the repair of CEI is more inherently challenging. Lee et al. discovered that the Li_6_PS_5_Cl_0.5_Br_0.5_‐based ASSLBs generate the C, S, Br, and O‐containing CEI species at the NCM/binder interface, which originate from the decomposition of SEs.^[^ ^145^ ^]^ Among these CEI species, the S‐ and C‐containing species can migrate and fill the microcracks in primary particles inside the secondary particles. Especially under pressure conditions, the macroscopic pores created by the volume‐variable are also reduced, this self‐healing mechanism of the CEI is essential for maintaining ionic transport (Figure 8D–F). Recently, the Li_1.3_Fe_1.2_Cl_4_ cathode with brittle to ductile transition and self‐healing behavior was reported by Fu et al., which facilitates superb cycling stability for 3000 cycles (90% capacity retention) at 5 C.^[^ ^146^ ^]^ This dynamic creeping cathode has excellent potential for maintaining the interface stability in SSLBs if combined with the self‐healing CEI.

**Figure 9:**
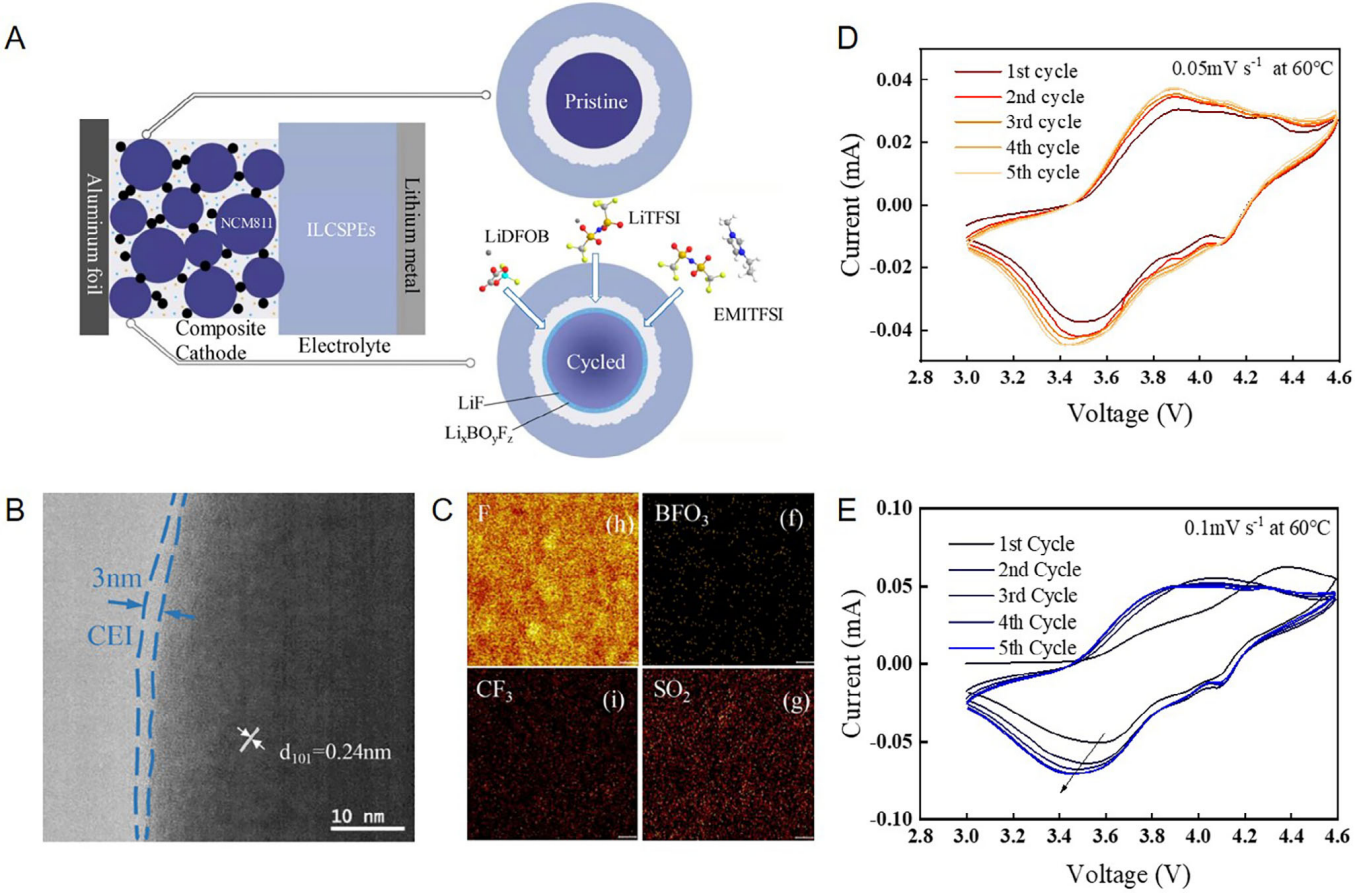
A) Schematic illustration of a dual‐anion for in situ construction CEI between the cathode and electrolyte in the SSLBs. B) TEM image of NCM cathode with LiDFOB after 100 cycles during 3.3–4.3 V. C) TOF‐SIMS analysis of F, BFO_3_, CF_3_, SO_2_ ions on NCM cathode with LiDFOB after 5 cycles at 0.1 C. D,E) Cyclic voltammetry of D) LiDFOB‐NCM|ILCPEs|Li and E) IL‐NCM|ILCPEs|Li cell.^[^ ^106^ ^]^ Copyright 2023, Elsevier.

Notably, the utilization of solid‐phase additives is also affected by the low fluidity of SEs, their limited dispersibility and fluidity pose challenges in not only inhibiting the uncontrollable growth of CEIs but also in forming stable, thin, and homogeneous CEIs. Furthermore, even the additives themselves are chemically unstable toward the SEs. Therefore, a more practical strategy is to introduce the additives directly in the composite cathode, exploiting the low fluidity of the solid phase to localize CEI formation on the cathode while avoiding any adverse effects on the anode or highly reactive SEs. The introduction of additives into the composite cathode can avoid the problem that the insoluble additives cannot be effectively utilized in SPE. Jiang et al. added low‐solubility LiDFOB to the composite cathode. These additives co‐decomposed with TFSI^−^ from PVDF‐HFP/ionic liquids electrolyte, yielding an in situ thin CEI layer consisting of LiF, Li_2_S_2_O_3,_ and Li_x_BO_y_F_z_. Consequently, this ultrathin CEI effectively suppresses the generation of oxidative peak in the initial cycle of cyclic voltammetry results above 4.3 V, while reducing charge‐transfer polarization, as well as accelerating interfacial lithium ion transport through the interface (**Figure** **9**A–E).^[^ ^106^ ^]^

**Figure 10:**
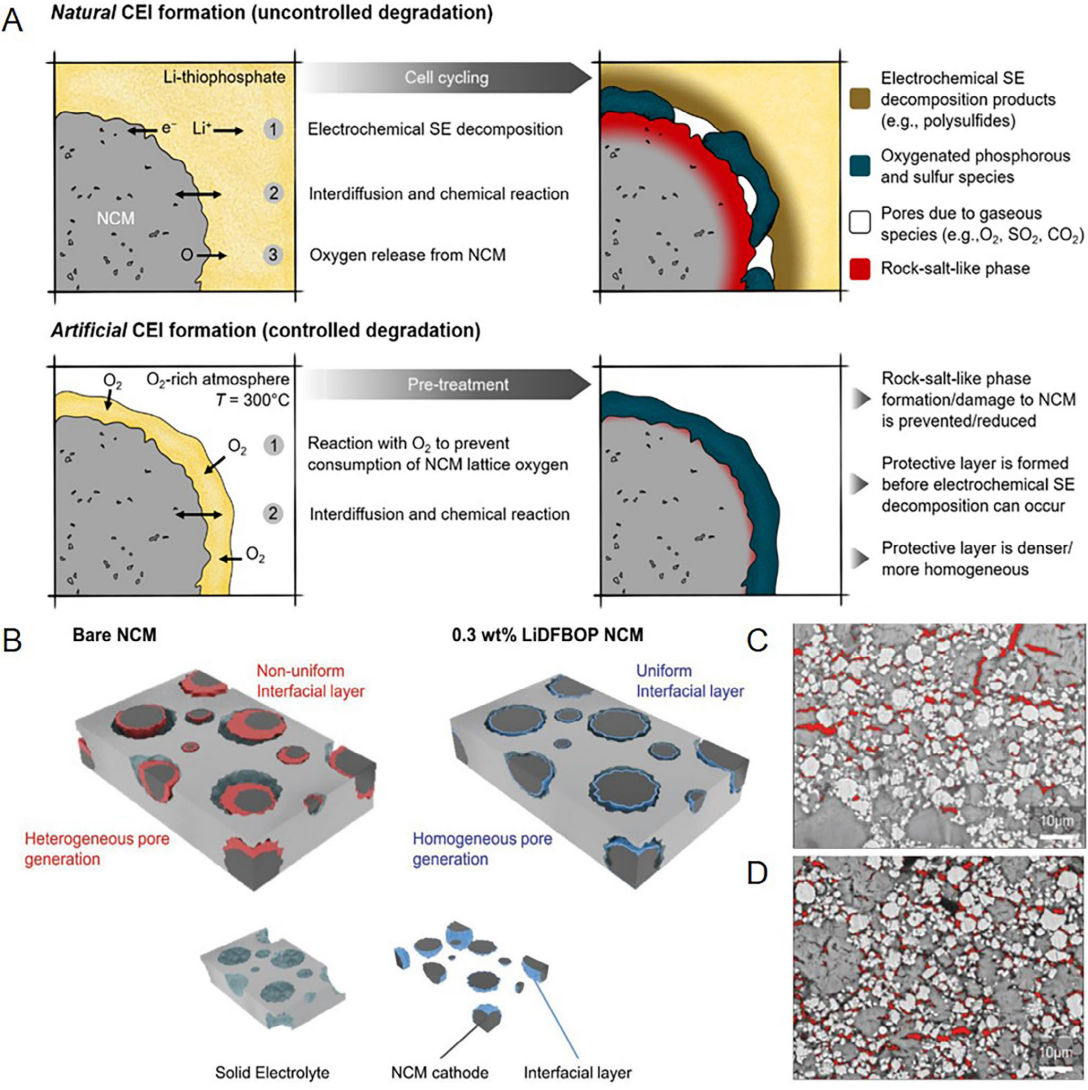
A) Schematic diagram of the natural and artificial CEI generation.^[^ ^57^ ^]^ Copyright 2023, American Chemical Society. B) Mechanism schematic illustration of CEI regulate chemical‐mechanical interface degradation between the cathode and electrolyte. C,D) Mean pore area ratio and pore size distribution for fresh materials, bare and NCM with 0.3 wt.%‐lithium difluorobis(oxalato)phosphate (LiDFBOP) after 100 cycles. Cross‐sectional SEM images of C) bare NCM and D) NCM with 0.3 wt.%‐LiDFBOP after 100 cycles.^[^ ^55^ ^]^ Copyright 2023, Wiley‐VCH.

Preserving the interfacial mechanical integrity are the prerequisite for long‐life SSLBs,^[^ ^55^ ^]^ as irreversible mechanical failure can destroy intimate cathode‐electrolyte contact and underline electrochemical degradation.^[^ ^68^ ^]^ Meanwhile, the interfacial decomposition cause the gas release (e.g., organic gas, O_2_, and H_2_) and the resulting open pores also sever the cathode‐electrolyte contact. Zuo et al. attempted to solve the abovementioned physical failures by pre‐coating NMC with Li_3_PS_4_ and LPSCl, then briefly heating the composite to trigger a controlled solid‐state reaction. This yields an artificial and stable CEI containing lithium‐phosphate/lithium‐sulfate under stress‐free and non‐electrochemical conditions rather than during cycling, effectively avoiding volume‐change‐induced cracks and contact loss.^[^ ^57^ ^]^ The resulting dense and uniform artificial CEI enables the Ni‐rich NMC|sulfide SSLB to retain 84% of capacity after 100 cycles within 2.6–4.5 V (**Figure** **10**A).

Contemporary sulfide‐ and halide‐based SSLBs are usually assembled with a composite cathode (high‐loading active material, SEs, and conductive carbon), SE membrane, and Li–In anode. The inevitable solid–solid contacts and physical gaps caused by cycling volume changes generate high interfacial impedance, critically hindering the electrochemical performance.^[^ ^147^ ^]^ Therefore, high stack pressure (ranging from several to hundred of MPa), an approach incompatible with practical packaging, is usually applied to ensure intimate interfacial contact. A practical route to SE deployment is CEI engineering that secures interfacial intimacy without resorting to high stack pressures. For example, Wan et al. paired F‐doped single‐crystal NMC811 with LPSCl and their interfacial compatibility could ensure the low‐impedance and crack‐resistant interface, which enabled 2.5 MPa operation.^[^ ^148^ ^]^ Park et al. applied the classical LiDFOB additive into a composite cathode via a mechanical blending process.^[^ ^55^ ^]^ This effectively inhibited the formation of detrimental sulfites or sulfates during cycling and promoted the formation of organics PO_3_‐ derived CEIs with a low Young's modulus. Furthermore, the reduced pores in the composite cathode enable ASSLBs to operate at a low stacking pressure of 0.75 MPa while maintaining favorable physical contact (Figure 10B–D).

### 5.2 Inferior Electrochemical Stability of SEs

**Figure 11:**
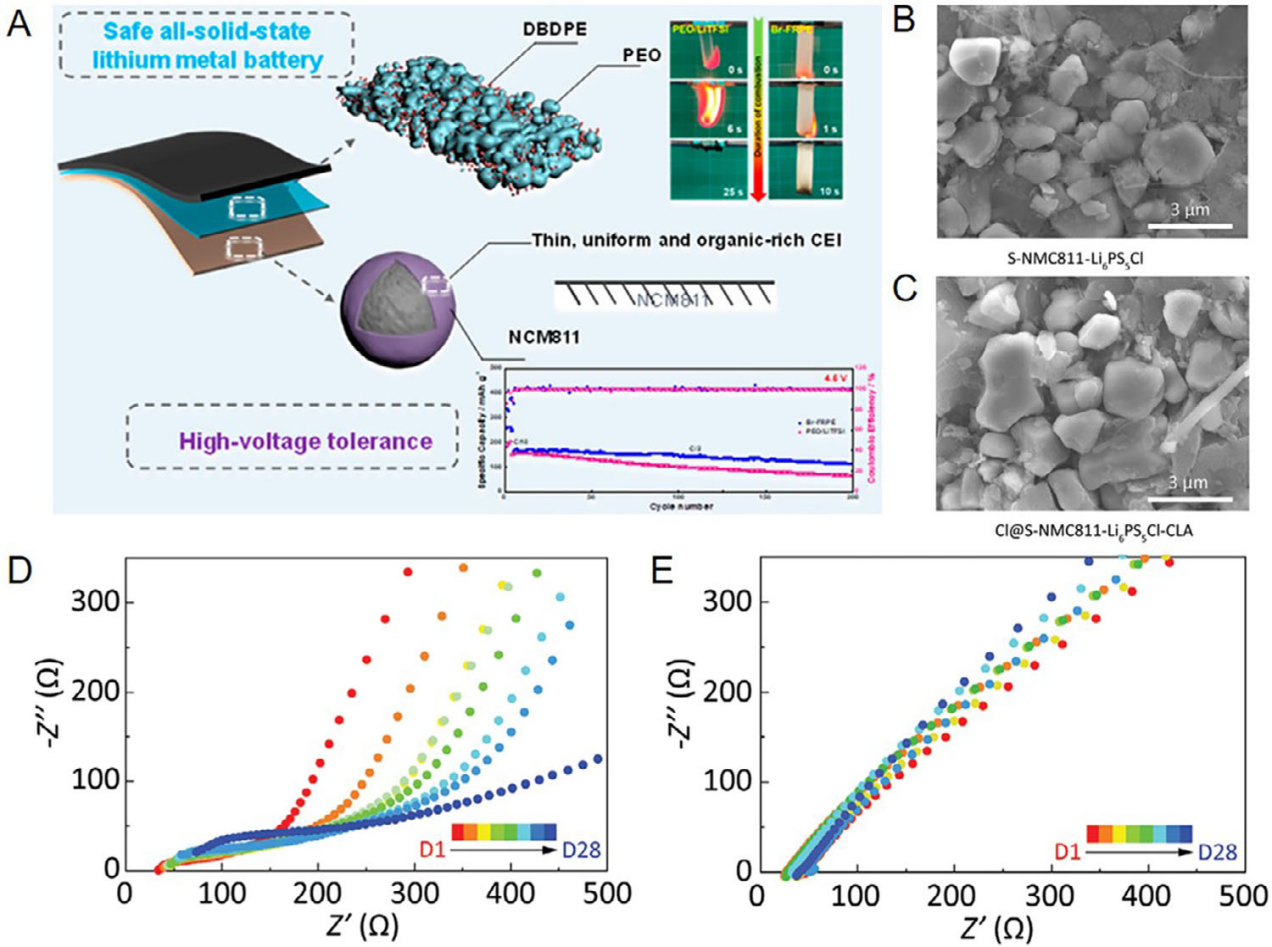
A) Design principles illustration of solid‐state electrolyte with flame‐retardant and high‐voltage tolerance in a safe and long cycling all‐SSLBs.^[^ ^124^ ^]^ Copyright 2022, American Chemical Society. B,C) SEM images of S‐NMC811‐Li_6_PS_5_Cl and Cl@S‐NMC811‐ Li_6_PS_5_Cl‐CLA cathode in ASSLBs after cycling. D,E) Impedance evolution of S‐NMC811‐Li_6_PS_5_Cl and Cl@S‐NMC811‐Li_6_PS_5_Cl‐CLA in ASSLBs under open‐circuit for 28 days.^[^ ^115^ ^]^ Copyright 2022, Wiley‐VCH.

SSLBs are designed for high energy density and are therefore routinely coupled with high‐voltage cathodes. However, the electrochemical windows of most SEs are limited, causing severe electrochemical instability at the interfaces between SEs and high‐voltage cathodes. To enhance the interfacial stability, it is critical to construct a robust CEI that facilitates efficient interfacial lithium ion transport. Such a CEI contributes significantly to the stability of both the cathode and the electrolyte, as evidenced by its role in mitigating element dissolution and structural degradation of cathode materials, as well as suppressing continuous electrolyte decomposition.^[^ ^72^ ^]^ Research on anions containing B─O bonds has been reported to utilize a coupling reaction that form anionic aggregates on the cathode surface, thereby enhancing interfacial stability.^[^ ^149^ ^]^ For instance, Han et al. employed LiDFOB to create a durable CEI with Li_x_B_x_O_y_ and LiF components, thereby securing LCO|PEO interfacial compatibility. As a result, the cell fabricated with modified PEO exhibits a significant improvement in capacity retention, increasing from 15% to 75% after 100 cycles.^[^ ^104^ ^]^ Interfacial compatibility also hinges on cathode chemistry. LFP‐based SSLBs can deliver stable electrochemical performance with SPEs due to the mild catalytic activity of Fe. However, TM‐based cathodes, such as LCO and LiNi_x_Mn_y_Co_1‐x‐y_O_2_, pose severe challenges in retaining the electrochemical stability with PEO. Their elevated cutoff voltages and the strong catalytic activity of Co, Ni, and Mn accelerate electrolyte oxidation and the side reactions between the PEO and the cathode,^[^ ^150^, ^151^ ^]^ causing the formation of thick CEIs with poor ionic conductivity and undermining long‐term stability (leading to a rapid decay in electrochemical performance).^[^ ^72^, ^152^ ^]^ Zhou et al. utilized the preferential oxidative decomposition of decabromodiphenyl ethane (DBDPE) in PEO to form LiBr at high‐voltage, inducing a uniform and organic/inorganic rich CEI on the NCM811.^[^ ^124^ ^]^ This CEI could facilitate the rapid diffusion of lithium ion across the interface and efficiently hinder further PEO oxidation. Consequently, the SSLBs with PEO‐based SPE and NCM811 could retain stable cyclability for 200 times with a cut‐off voltage of 4.5 V (**Figure** **11**A).

**Figure 12:**
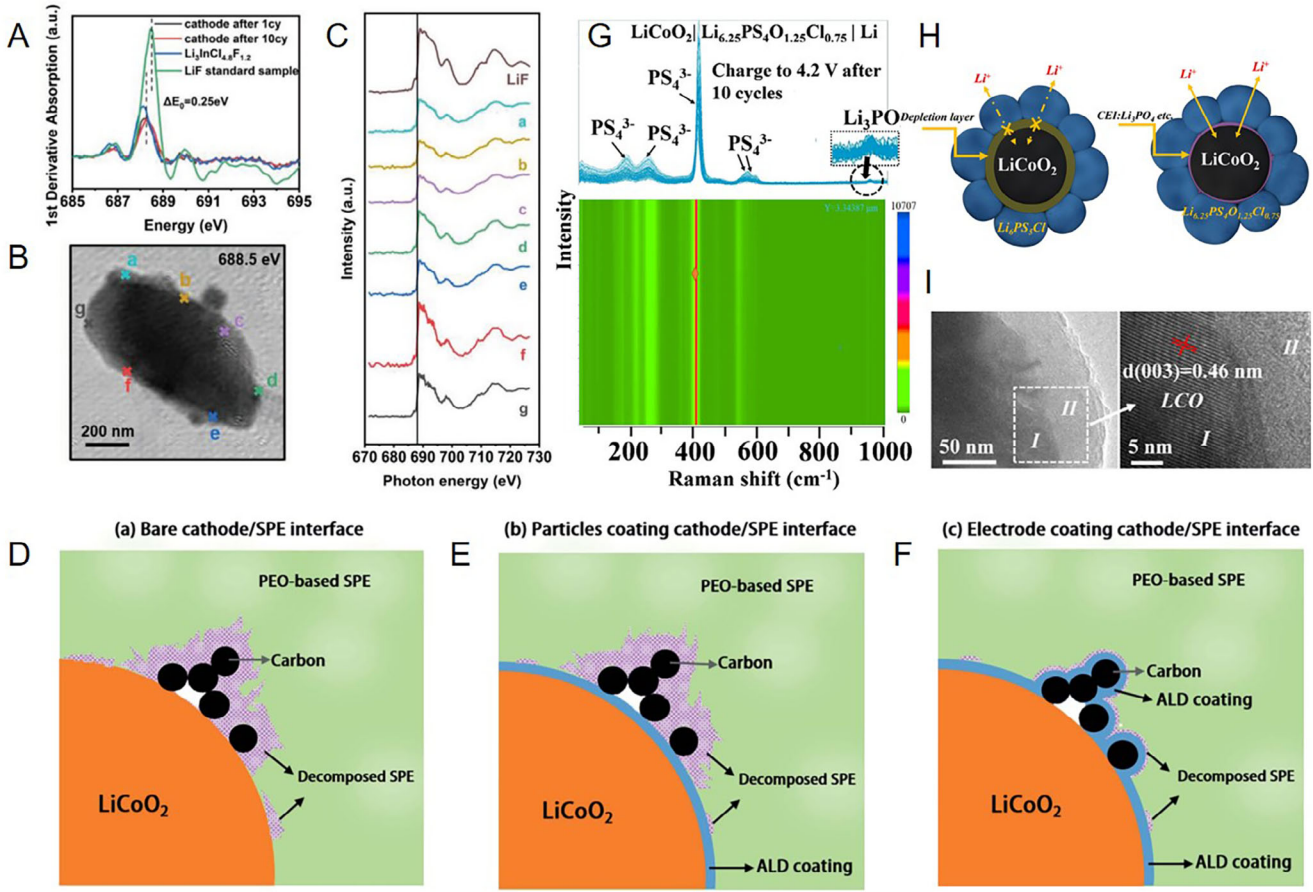
A) First derivative absorption spectra of synchrotron spectroscopic for standard LiF, Li_3_InCl_4.8_F_1.2_, and LCO|Li_3_InCl_4.8_F_1.2_ composite cathode after 1st and 10th cycles. B) X‐ray ptychography of a single LCO particle after 50 cycles at 688.5 eV. C) F K‐edge XAS spectrum compared with that of the standard LiF at different marked positions.^[^ ^71^ ^]^ Copyright 2021, Wiley‐VCH. D–F) Mechanism schematic illustration of ALD coating for enabling stable and high‐voltage tolerance for solid polymer electrolyte‐based batteries.^[^ ^73^ ^]^ Copyright 2023, Royal Society of Chemistry. G) Raman spectra of the generated CEI at the LCO|Li_6.25_PS_4_O_1.25_Cl_0.75_ interface of ASSLBs. H) Schematic diagram for the depletion layer generated and in situ generated Li_3_PO_4_ at the interface of LCO|Li_6_PS_5_Cl. I) HRTEM patterns of the CEI originated from Li_6.25_PS_4_O_1.25_Cl_0.75_ coated on LCO and the corresponding magnified regions.^[^ ^67^ ^]^ Copyright 2022, Wiley‐VCH.

The narrow electrochemical window of sulfide SEs renders them intrinsically incompatible with high‐voltage cathodes.^[^ ^113^ ^]^ Oxidative instability of the S^2−^ anion triggers persistent side reactions at the cathode‐electrolyte interface, yielding unstable CEI and causing rapid capacity fade, especially at high‐voltage above 4 V.^[^ ^153^, ^154^ ^]^ Although the coating strategies, such as Li_2_ZrO_3_ coating, can slow interfacial side reactions, these inorganic coating layers neither suppress transition metal migration nor mitigate the electronic resistance rise, and their rigidity often make them to crack under anisotropic cathode expansion.^[^ ^155^, ^156^, ^157^, ^158^, ^159^ ^]^ Therefore, modifying the cathode/electrolyte materials may represent an effective method to relieve side reaction and improve CEI at the cathode‐electrolyte interface. Wan et al. developed a single‐crystalline cathode NMC811 by doping Cl, which enhances cathode stability and avoids cracks of the materials, benefiting CEI intact. Moreover, a small amount of AlF_3_ (0.02 wt.%) additives were added into LPSCl SEs to further facilitate the formation of F‐containing CEI with high oxidative stability. Consequently, the good compatibility of Cl‐doped single‐crystalline‐NMC811 mixed with Li_6_PS_5_Cl and CuF_2_‐LiNO_3_‐AlF_3_ (Cl@S‐NMC811‐Li_6_PS_5_Cl‐CLA) exhibits more stable EIS spectra compared to the rapidly increased impedance of single‐crystalline‐NMC811 mixed with Li_6_PS_5_Cl (S‐NMC811‐Li_6_PS_5_Cl) in ASSLBs under open‐circuit conditions. Hence, sulfide‐based ASSLBs successfully achieve enhanced stability and exhibit a capacity retention of 69.4% after 100 cycles with an areal capacity of 2.55 mAh cm^−2^ (Figure 11B–E).^[^ ^115^ ^]^ The interface stability at ultra‐high‐voltage in ASSLB have been studied quantitatively. Zhang et al. investigated the interface between LCO and Li_3_InCl_4.8_F_1.2_ at 4.8 V and found that the LiF‐rich CEI generated on the LCO surface.^[^ ^71^ ^]^ The Li_3_InCl_4.8_F_1.2_ exhibits low decomposition energy against LCO at lithiation and delithiation state (−15 and −7 meV, respectively), indicating a readily decomposition reaction of SEs. In contrast, LiF demonstrates a 0 meV of decomposition energy against LCO, significantly improving the reaction barrier and stabilize the cathode/electrolyte interface at high‐voltage (**Figure** **12**A–C).

In LPSCl‐based SSLBs, the elements between the LCO cathode and LPSCl can diffuse with each other, causing the interfacial degradation and thus forming the electronically conductive cobalt sulfide during cycling.^[^ ^160^ ^]^ Consequently, the harmful passivation layer gradually grows and become thick, inevitably increasing the interfacial resistance. To suppress the formation of conductive cobalt sulfide in LPSCl‐based SSLBs, some effective strategies have been conducted, such as substitution of electrochemically active P─S bonds, surface coating of cathode materials, surface coating of electrolyte materials, and introduction of SEs additives. Kim et al. introduced 2‐(trimethylsilyl)ethanethiol in LE as a high‐voltage tolerance additive, which possesses thiol functional groups that could be uniformly and thinly adsorbed on the surface of LPSCl electrolyte.^[^ ^116^ ^]^ It significantly inhibited the formation of conductive cobalt sulfide, improved the conductivity of CEI and the passivation layer, and restrained the continuous growth of CEI and interface resistance. As a result, the batteries could be cycled for 2000 cycles and retain 85% capacity.

Beyond the interfacial issue discussed above due to the instability of cathodes and electrolytes, carbon conductive additives also significantly undermine interfacial stability. High‐surface‐area carbon provides abundant catalytic sites that drive parasitic electron transfer, rendering SEs highly reactive with conductive phases and accelerating interfacial degradation. Therefore, isolating SEs from these electron pathways is critical. To enhance the battery performance, Sun et al. employed a strategy of coating lithium titanate on the Super‐P surface using ALD technology. This approach protected the carbon/SPE interface, which resulted in the PEO‐based batteries with superior stability and cycling performance, even when using a highly reactive LCO cathode at 4.5 V (Figure 12D–F).^[^ ^73^ ^]^ In term of SE materials modification, Xu et al. enhanced the chemical stability at the LPSCl and LCO contact by performing O‐doping electrolyte. The formed Li_3_PO_4_ inorganic oxides enhanced the electronic insulating ability of CEIs, inhibiting the generation of ${\mathrm{SO}}_{3}^{2-}$ and P_2_S_5_ phases (Li depletion phase of LPSCl) and controlling the growth of CEIs (Figure 12G–I).^[^ ^67^ ^]^

### 5.3 Poor Transport Kinetics of Li Ions

**Figure 13:**
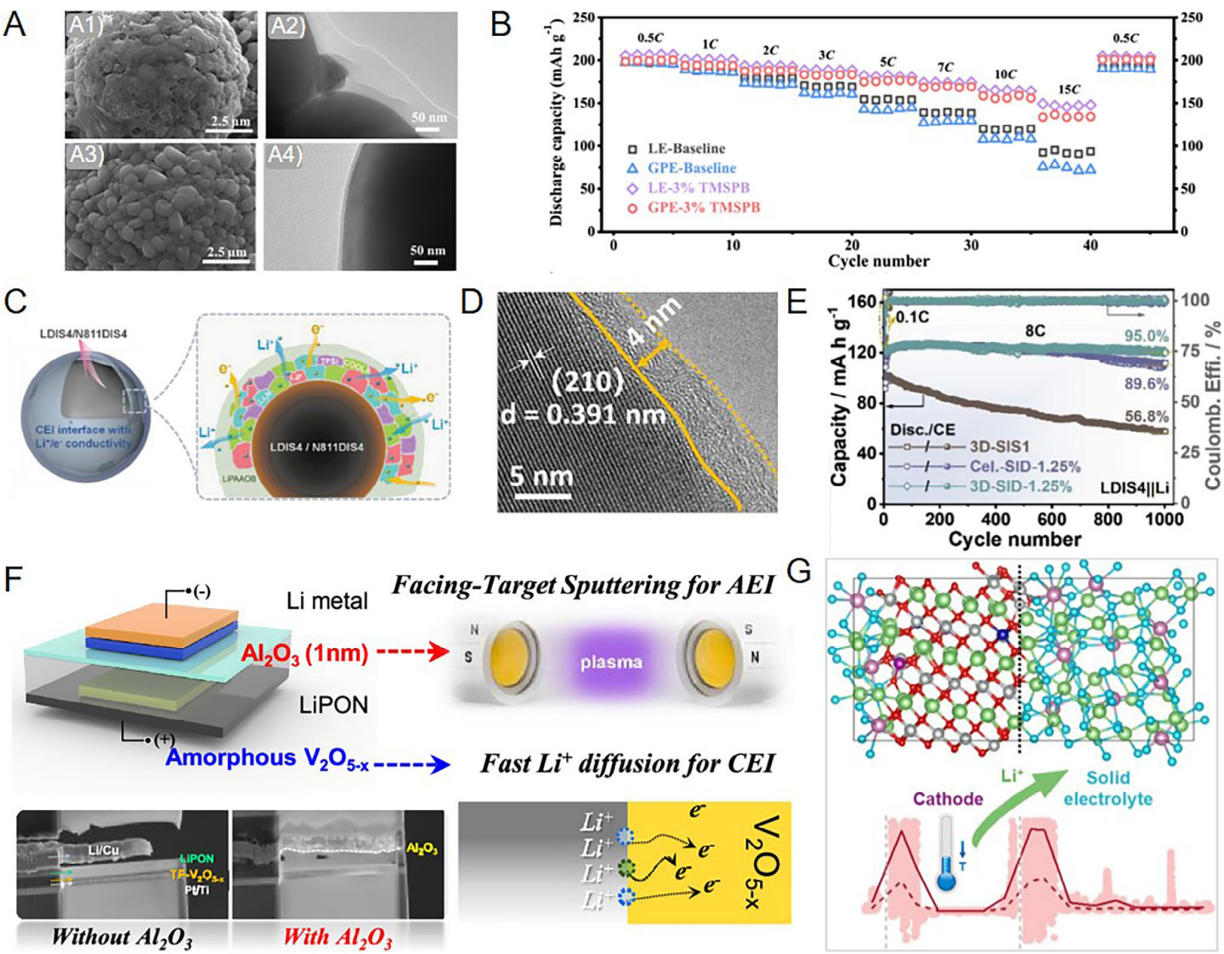
A) SEM and TEM images of NCM811 in A1,A2) LE‐Based, A3,A4) GPE electrolytes after 100 cycles. B) Rate performance of Li||NCM811 batteries with various electrolytes.^[^ ^86^ ^]^ Copyright 2022, American Chemical Society. C) Structure illustration of conductive CEI layer of cathode particles after cycling. D) HRTEM image of dual salt‐optimized LFP electrode (LDIS4). E) Long‐term cycling performances LDIS4||Li based on various electrolyte at 8 C.^[^ ^62^ ^]^ Copyright 2023, Wiley‐VCH. F) Schematic diagrams of fabricated thin‐film solid‐state batteries with a protective ultrathin Al_2_O_3_ layer, LiPON electrolyte, and a V_2_O_5−x_ cathode.^[^ ^120^ ^]^ Copyright 2021, American Chemical Society. G) Theoretical evaluation of interfacial energy barrier.^[^ ^163^ ^]^ Copyright 2024, American Chemical Society.

Although significant advances have been made in the ionic conductivity of SEs, the overall kinetic properties of SSLBs remain unsatisfactory for practical applications.^[^ ^161^ ^]^ The GPEs and sulfide electrolytes exhibit ionic conductivities comparable to those of LEs, but their poor interfacial wettability and tendency to undergo side reactions significantly impair the kinetic performance of SSLBs. Particularly at electrode/electrolyte interfaces, interface polarization, including inferior physical contact, side reactions, and the space charge layer, significantly hinder the ionic transport kinetic of SSLB.^[^ ^162^ ^]^ These kinetic limitations are even more pronounced in less conductive electrolytes such as SPEs and OEs. To address these challenges, the CEI must be both highly conductive to lithium ion and sufficiently thin. For instance, Li et al. introduced 3‐(trimethylmethylsilyl)phenylboronic acid (TMSPB) as an additive into PE‐PVDF‐HFP‐based GPE,^[^ ^86^ ^]^ leading to B‐containing CEI with a thickness of ≈10 nm on the NMC cathode surface. The B─O bond could facilitate lithium ion migration and then decrease the interfacial impedance, enabling GPE‐based SSLB to retain a capacity of 133.5 mAh g^−1^ at 15 C (**Figure** **13**A,B). Similarly, Hu et al. have developed a GPE with a 3D cross‐linked network containing ─C≡N groups, which promotes the formation of an ultrathin (3–6 nm) and high‐quality CEI. This CEI is thinner than that formed in conventional polypropylene‐LiPF_6_‐EC/DMC‐based LIBs, significantly enhancing lithium ion migration across LiFePO_4_|electrolyte interphase. Additionally, the ─C≡N groups enhance the lithium ion transference efficiency by strong coordination with the lithium ion and electrostatic repulsion with the PF_6_^−^ anion. Consequently, the LiFePO_4_||Li SSLBs deliver a capacity of 106 mAh g^−1^ and maintain 1000 cycles at 6 C.^[^ ^93^ ^]^

The functionality of the CEI layer also plays a critical role in determining kinetic performance. Chai et al. have reported that a supramolecular lithium polyacrylic acid oxalate borate (LiPAAOB) conductor salt can induce molecular interactions within GPEs. This additive not only supplies additional lithium ion to promote the dissociation of LiTFSI but also forms a stable coordination structure with the polymer matrix via strong hydrogen‐bonding interactions. The designed GPE allows the construction of an artificial CEI as thin as 4 nm, which is composed by ion‐conducting Li_3_N and LiF. Along with this, a single ion conductor polymer replaces the insulating PVDF that result in impressive electronic conductivities of 28.3 × 10^−2^ mS cm^−1^, thus forming a dual lithium ion/electron conducting interface. The corresponding NMC811‐based SSLBs exhibit a good rate performance at 8 C and maintain 1000 cycles (Figure 13C–E).^[^ ^62^ ^]^ However, it should be noted that excessive use of additives can lead to thick CEI layers, which increase interfacial impedance and degrade battery performance.^[^ ^97^ ^]^

Regulating the intrinsic properties of cathode materials is regarded as an effective strategy to enhance the kinetic performance of CEI. Xiao et al. utilized the well‐defined O vacancy sites in amorphous V_2_O_5−x_ cathodes to facilitate isotropic lithium ion diffusion in CEI and improve both ionic/electronic conductivities. These abundant sites effectively prevent the lithium ion detour and reduce the volume change between the cathode and LiPON during cycling. The thin‐film batteries deliver a 474.01 mA cm^−3^ for the 400th cycle (Figure 13F).^[^ ^120^ ^]^ Moreover, engineering the interfacial architecture and electrolyte composition to facilitate superior ionic transport kinetics across the CEI represents another promising strategy for performance optimization. Lu et al. inserted a Li_2_ZrO_3_ coating layer to modulate the interface between Ni90 and LPSCl sulfide SEs, reducing the interfacial activation energy from 60.19 to 41.39 kJ mol^−1^. Further replacement of LPSCl with Li_3_InCl_6_ results in an ultralow interfacial activation energy of 25.79 kJ mol^−1^, suggesting the CEI induced by materials properties notably enhance interfacial lithium ion transport. This improved CEI design realizes an enhanced capacity retention (26.9%) at −30 °C in Ni90|Li_3_InCl_6_|LPSCl|Li‐In all‐solid‐state batteries (Figure 13G).^[^ ^163^ ^]^

**Figure 14:**
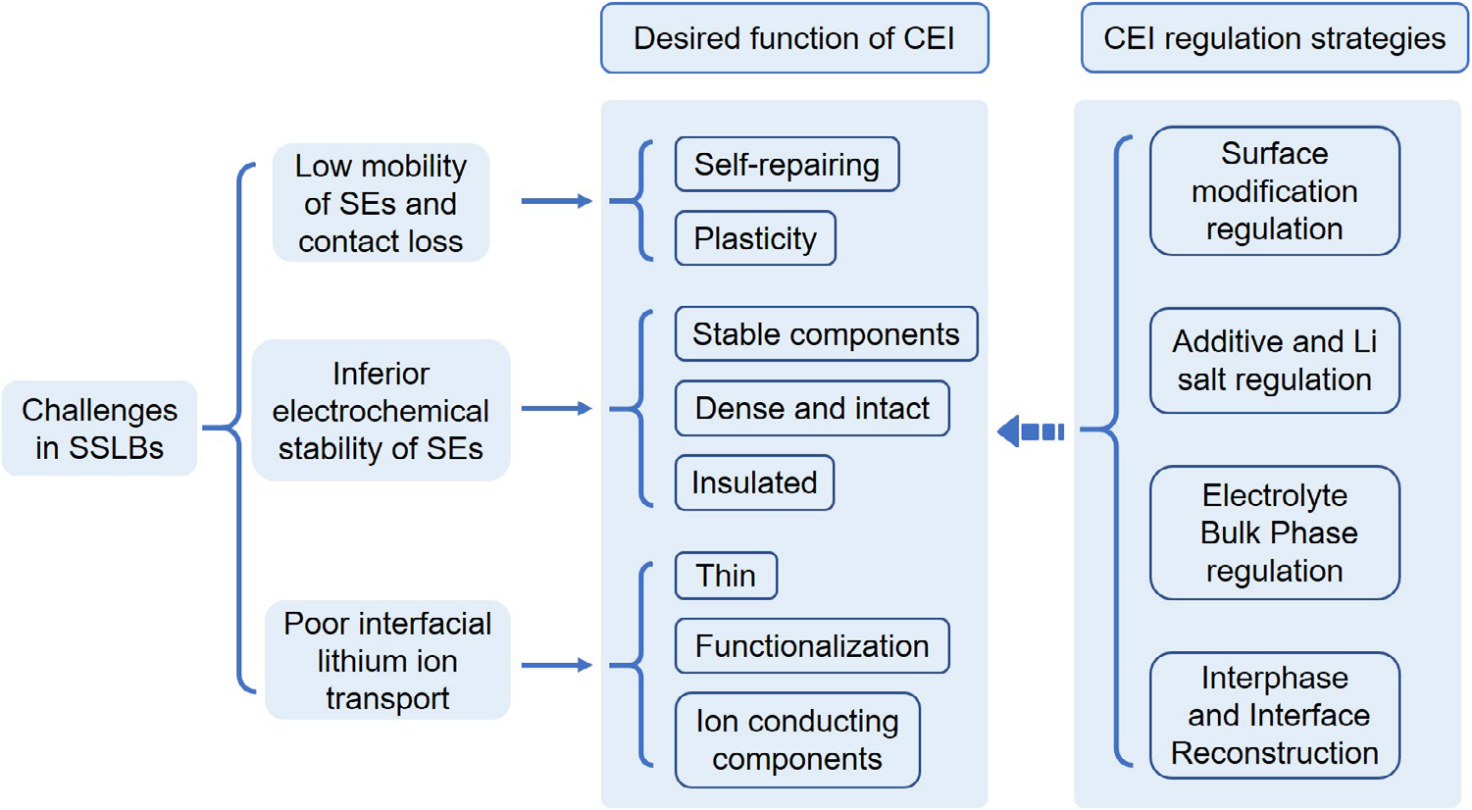
Regulation strategies and expected function of CEI for addressing challenges in SSLBs.

In SSLBs, CEI plays a crucial role in addressing key challenges, including the low mobility of SEs and contact loss, the inferior electrochemical stability of SEs, and poor interfacial lithium ion transport. As shown in **Figure** **14**, the desired functionality of the CEI requires self‐healing capabilities and plasticity to mitigate issues of low electrolyte mobility and physical contact loss. To address insufficient electrochemical stability, stable CEI components, dense and intact interphase structures, and electronic insulation are essential. Furthermore, thin and functionalized CEI layers incorporating ion‐conducting components are critical for enhancing interfacial ion transport. Importantly, realizing the diverse capabilities of CEI demands integrated application of multiple regulation strategies rather than relying on any single approach, ultimately leading to improved battery performance, stability, and cycle life.

## 6 Conclusion and Perspectives

Solid‐state lithium batteries are advancing toward higher energy density, propelled by the adoption of high‐capacity and high‐voltage cathodes. Nevertheless, formidable interfacial challenges at the cathode side remain critical bottlenecks. Understanding the essential role of the CEI and constructing a robust interface is indispensable for high‐energy‐density and long‐life SSLBs. Although the CEIs in SSLBs, particularly in systems employing organic electrolytes, retain composition and functionality similarities with that in liquid LIBs, such CEIs in SSLBs exhibit unique degradation mechanisms, including physical contact loss, pronounced volume variations, and restricted CEI self‐healing that compromise cathode‐electrolyte interfacial stability. These challenges are further exacerbated in SSLBs utilizing inorganic solid electrolytes, where CEI compositions vary considerably across halide, sulfide, and oxide electrolytes due to distinct material and by‐products. Another distinction between SSLBs and liquid LIBs lies in the physical extent and conceptual definition of the CEI. Traditional CEI concepts focus narrowly on nanoscale decomposition layers on the cathode surface. In SSLBs, the immobility of SEs enables electrochemical by‐products to precipitate across the entire electrolyte surface and even convert the bulk electrolyte into a passivation layer. Therefore, we propose a generalized CEI definition that embraces all interphases, including interfacial parts and bulk electrolyte passivation layer, created by cathode‐electrolyte interactions. Practical limitations of SEs, such as low mobility, electrochemical instability, physical contact loss, and poor ion kinetics, collectively compromise CEI self‐healing, additive utilization, compositional control, electrolyte stability, and lithium ion transport dynamics. Current research strategies, including surface modification, functional additives, and Li salt design, electrolyte regulation, and interphase reconstruction, are actively pursued to address these interfacial challenges, along with the expectation of constructing highly stable and lithium ion‐conductive CEI.

High‐voltage cathode species, including NCM, LCO, LiNi_0.5_Mn_1.5_O_2_ (LNMO), and xLi_2_MnO_3_⋅(1−x)LiTMO_2_ (LLO), have reached a high cutoff voltage above 4.6 V and even 4.8 V, demanding CEIs with exceptional oxidation stability and electron insulation. Consequently, CEI design in SSLBs prioritizes inorganic‐rich components since organics cannot withstand such high‐voltage and oxidize rapidly. However, higher inorganic content increases CEI brittleness, underscoring the importance of an optimal organic–inorganic ratio to accommodate volume changes while preserving high‐voltage durability.

Most SEs exhibit lower ionic conductivity than LEs, and poor solid‐solid interfacial contact further leads to high interfacial impedance, lowing electrochemical capacity. Therefore, constructing the CEI with suitable thickness, high lithium ion conductivity, and effective electronic insulation is essential for improving the rate capability of SSLBs. The ideal CEI thickness must strike a balance: it should be sufficiently thick to stabilize the cathode structure and suppress continuous electrolyte decomposition, yet not excessively thick to avoid high impedance for lithium ion transport. Given the various combinations of SEs and cathodes, the structure, thickness, and composition of the CEI can vary significantly. It is vital to develop a fundamental understanding of the lithium ion transport mechanisms and multi‐scale lithium ion dynamics within the CEI and CEI‐like buffer layer across various SE systems.

As outlined in the extended definition of CEI, the SLEI and CEI‐like buffer layer also contribute significantly to stabilizing the cathode‐side interface, meriting deeper mechanistic investigation. For instance, in GPE and SPE systems, research has predominantly focused on CEI formation directly on the cathode surface, while degradation processes at the electrolyte side and the role of SLEI remain considerably underexplored. In the sulfide/hydride system, CEI‐like buffer layers exhibit complex compositions. The functions of their individual components and formation mechanisms are still unclear, demanding urgent and in‐depth study. Furthermore, the targeted regulation and modification of such CEI‐like interphases represent a promising avenue for enhancing cathode‐side interfacial stability, offering a valuable direction for future research.

The development and application advanced characterization techniques are also essential to elucidate complex interfacial phenomena and underlying mechanisms in SSLBs. The corresponding tools, such as in situ electrochemical testing, in situ spectroscopy, and high‐resolution cryo‐electron microscopy, enable atomic‐scale observation of the chemical and structural evolution within CEIs, beneficial for constructing durable CEI and developing interfacial chemistry in SSLBs. We believe that these insights in this review will deepen fundamental understanding of CEI and promote the development of high‐energy‐density SSLBs.

## Conflict of Interest

The authors declare no conflict of interest.
